# LincRNA H19 protects from dietary obesity by constraining expression of monoallelic genes in brown fat

**DOI:** 10.1038/s41467-018-05933-8

**Published:** 2018-09-06

**Authors:** Elena Schmidt, Ines Dhaouadi, Isabella Gaziano, Matteo Oliverio, Paul Klemm, Motoharu Awazawa, Gerfried Mitterer, Eduardo Fernandez-Rebollo, Marta Pradas-Juni, Wolfgang Wagner, Philipp Hammerschmidt, Rute Loureiro, Christoph Kiefer, Nils R. Hansmeier, Sajjad Khani, Matteo Bergami, Markus Heine, Evgenia Ntini, Peter Frommolt, Peter Zentis, Ulf Andersson Ørom, Jörg Heeren, Matthias Blüher, Martin Bilban, Jan-Wilhelm Kornfeld

**Affiliations:** 1 Max Planck Institute for Metabolism Research, Gleueler Strasse 50, 50931 Köln, Germany; 2 Cologne Cluster of Excellence: Cellular Stress Responses in Ageing-associated Diseases (CECAD), Joseph-Stelzmann-Str. 26, 50931 Köln, Germany; 3 Department of Laboratory Medicine, Medical University of Vienna, 1090 Vienna, Austria; 4 Department for Biochemistry and Molecular Biology (BMB), University of Southern Denmark, Campusvej 55, 5230 Odense M, Denmark; 5 Helmholtz-Institute for Biomedical Engineering, Stem Cell Biology and Cellular Engineering, RWTH Aachen University Medical School, Pauwelstrasse 20, 52074 Aachen, Germany; 6 Institute for Biochemistry and Molecular Cell Biology, Martinistraße 52, 20246 Hamburg, Germany; 7 Max Planck Institute for Molecular Genetics, Ihnestrasse 63-73, 14195 Berlin, Germany; 8 Institute for Molecular Biology and Genetics, Aarhus University, C F Møllers Alle 3, 8000 Aarhus C, Denmark; 9 Department of Medicine, University of Leipzig, 04103 Leipzig, Germany; 10 Core Facilities, Medical University of Vienna, 1090 Vienna, Austria

## Abstract

Increasing brown adipose tissue (BAT) thermogenesis in mice and humans improves metabolic health and understanding BAT function is of interest for novel approaches to counteract obesity. The role of long noncoding RNAs (lncRNAs) in these processes remains elusive. We observed maternally expressed, imprinted lncRNA *H19* increased upon cold-activation and decreased in obesity in BAT. Inverse correlations of *H19* with BMI were also observed in humans. *H19* overexpression promoted, while silencing of *H19* impaired adipogenesis, oxidative metabolism and mitochondrial respiration in brown but not white adipocytes. In vivo, *H19* overexpression protected against DIO, improved insulin sensitivity and mitochondrial biogenesis, whereas fat *H19* loss sensitized towards HFD weight gains. Strikingly, paternally expressed genes (PEG) were largely absent from BAT and we demonstrated that *H19* recruits PEG-inactivating *H19*-MBD1 complexes and acts as BAT-selective PEG gatekeeper. This has implications for our understanding how monoallelic gene expression affects metabolism in rodents and, potentially, humans.

## Abstract (web summary)

Brown adipose tissue (BAT) thermogenesis counteracts obesity and promotes metabolic health. The role of long non-coding RNAs (lncRNAs) in the regulation of this process is not well understood. Here the authors identify a maternally expressed lncRNA, H19, that increases BAT oxidative metabolism and energy expenditure.

## Introduction

Obesity results from chronic imbalances between caloric intake and energy expenditure (EE), which finally culminates in development of cardiometabolic complications^1^, artherosclerosis,^2^ liver manifestations of metabolic disease^3^, and type 2 diabetes (T2D)^1^. In contrast to white adipose tissue (WAT), which primarily stores lipids, brown adipose tissue (BAT) represents a specialized organ that supports energy catabolism by converting dietary and stored nutrients like lipids and glucose into heat (non-shivering thermogenesis) by virtue of uncoupling the electrochemical energies stored in proton gradients across mitochondrial membranes^4^. The (re)-discovery of functional BAT in (adult) humans^5^, the observation that surgical and genetic ablation of BAT sensitizes towards obesity in mice^6^ and the clinically relevant fact that reductions in ambient temperatures improve metabolism in lean^7^ and diabetic^8^ subjects, spurred great interest in harnessing the catabolic properties of BAT for therapeutic purposes. In recent years, significant progress was made in understanding and functionally dissecting the transcriptional and epigenetic control of BAT differentiation and function by protein-coding genes^9^. Yet, large-scale sequencing initiatives such as ENCODE^10^ demonstrated that RNA transcription is not confined to protein-coding genes but constitutes a pervasive phenomenon observed throughout the majority of genomes in higher organisms^11^, giving rise to thousands of small noncoding RNAs like microRNAs but also long noncoding RNAs (lncRNAs). LncRNAs are (arbitrarily) defined as transcripts ≥200 nt with low species conservation at the nucleotide level, low coding potentials, and simplistic gene architectures^12^. Although lncRNA numbers are debated, RNA-Seq meta-analyses in human tissues identified >60,000 lncRNAs, which surpassed the ca. 22,000 coding genes detected in the same study^13^.

Despite progress in identifying and functionally dissecting lncRNAs involved in cellular and organismal ageing^14^, metabolic homeostasis^15^, and adipose tissue biology^16,17^, our understanding of how lncRNA control adipogenesis and BAT differentiation and function in particular remains elusive. To gain molecular insights into these processes, we here performed RNA-Sequencing (RNA-Seq) in BAT of mice exposed cold or exposed to chronic high-fat diet feeding and observed that monoallelically expressed (imprinted) lncRNA *H19* correlated with BAT activation in mouse, but also humans. In vitro and in vivo gene manipulation identified BAT-selective roles for *H19* in controlling adipocyte differentiation and function and systemic energy metabolism. Crucially, we found that many adipose-selective imprinted genes expressed from paternal alleles were absent from BAT and could demonstrate that *H19* forms *H19*-MBD1 chromatin modifier complexes that specifically repress paternally expressed imprinted genes in brown, not white, adipocytes, thereby serving as selective PEG gatekeeper in BAT.

## Results

### RNA-sequencing for BAT-regulatory lncRNAs

**Fig. 1:**
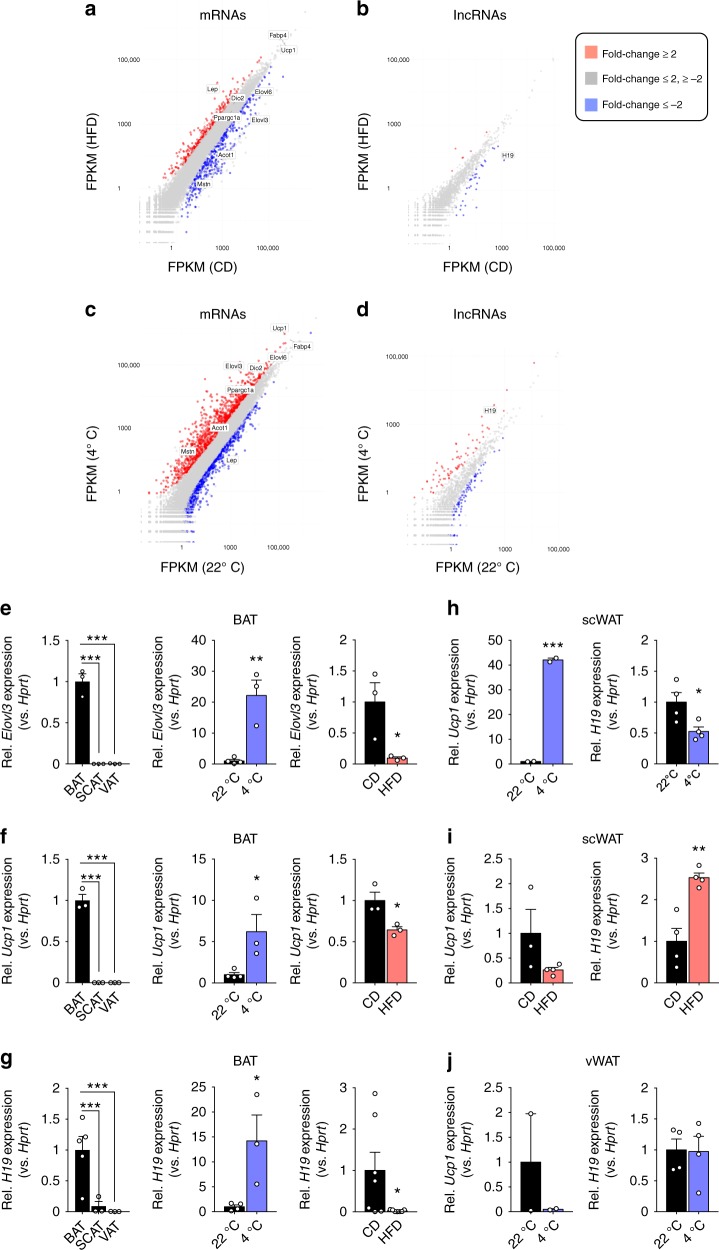
RNA-Seq reveals lincRNAs correlating with brown fat function in vivo. **a**, **b** Plot of BAT (**a**) mRNA or (**b**) lincRNA expression after HFD or CD feeding (RNA-Seq, *n* = 2 per biological condition). **c**, **d** Plot of BAT **c** mRNA or **d** lincRNA expression after 24 h of 4 °C or 22 °C (RNA-Seq, *n* = 2 per biological condition). **e**–**g** BAT, scWAT, and vWAT expression of **e** *Elovl3* **f** *Ucp1*, and **g** *H19* expression in CD-fed mice (*n* = 3–5 per given tissue, left), in CD-fed mice housed at 22 °C (*n* = 4) versus 24 h of 4 °C (*n* = 3, middle) and HFD-fed (*n* = 3 for *Elovl3* and *Ucp1*, *n* = 7 for *H19*) versus CD-fed mice (*n* = 3 for *Elovl3* and *Ucp1*, *n* = 7 for *H19*, right, qRT-PCR. **h**, **i** scWAT expression of *Ucp1* and *H19* in **h** mice housed at 22 °C (*n* = 2 for *Ucp1*, *n* = 4 for *H19*) versus 24 h of 4 °C (*n* = 2 for *Ucp1*, *n* = 4 for *H19*) and **i** HFD-fed (*n* = 4) versus CD-fed (*n* = 3 for *Ucp1*, *n* = 4 for *H19*) mice. **j** vWAT *Ucp1* and *H19* expression in mice housed at 22 °C versus 24 h of 4 °C (*n* = 2 for *Ucp1*, *n* = 4 for *H19*). Unless stated, bar graphs represent mean ± s.e.m. with all data points plotted and unpaired, two-tailed Student’s *t*-tests were used to assess statistical significance. *p < 0.05, **p < 0.01, ***p < 0.001. If applicable *p*-values are indicated within the panel

To identify mRNAs and lncRNAs correlating with BAT function, we exposed lean C57BL/6 mice to thermal stress (4 °C for 24 h) compared to 22 °C housed mice or to chronic high-fat-diet (HFD) compared to micronutrient-matched control diet (CD) feeding. Next, we performed RNA-Sequencing (RNA-Seq) in these BAT samples and observed 1394/433 upregulated and 1147/429 downregulated mRNAs together with 71/6 upregulated and 101/33 downregulated lncRNAs across cold/DIO conditions (fold-change ≥2 or ≤−2; Fig. 1a-d, Supplementary Data 1,2). Alterations in BAT thermogenesis were confirmed by elevated and decreased *Uncoupling Protein 1 (Ucp1)* and *ELOVL Fatty Acid Elongase 3* (*Elovl3*) mRNA expression during cold or diet-induced obesity (DIO), respectively (Fig. 1e, f). Due to its BAT-selective expression—as for *Ucp1* and *Elovl3* (Fig. 1e–g)—and strong eutherian sequence conservation, we focussed on the intergenic lncRNA (lincRNA) *H19*, which was induced in cold-exposed and decreased in obese BAT (Fig. 1g). Notably, *H19* expression was not altered accordingly in subcutaneous (scWAT) or visceral (vWAT) white adipose depots in contrast to browning/beiging markers like *Ucp1* (Fig. 1h-j), arguing for a selective involvement of *H19* in brown but not beige adipocyte differentiation and function.

### H19 controls brown adipogenesis and BAT oxidative metabolism

**Fig. 2:**
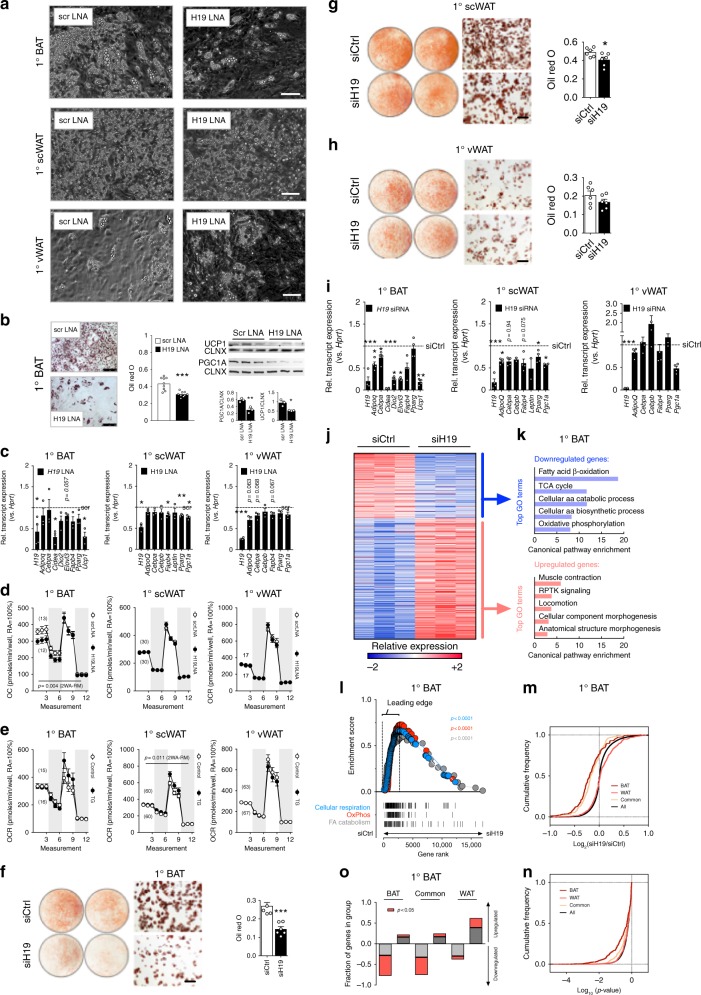
LincRNA H19 required for brown but not white adipocyte differentiation and function. **a** Photomicrograph of BAT, scWAT, and vWAT 1° adipocytes transfected with scrambled (scr) or *H19* LNAs. Pictures represent *n* = 6 (1°BAT) or *n* = 3 (others) experiments, *n* = 3 replicates each. Scale bar, 100 µm **b** ORO staining and quantification, PGC1A/UCP1 immunoblot and quantification of 1°BAT transfected with scr or *H19* LNA. Scale bar, 250 µm. **c** Expression of indicated mRNAs in BAT, scWAT and vWAT 1° adipocytes transfected with scr or *H19* LNAs. A paired, two-tailed Student’s *t*-test was used to assess significance across *n* = 3 experiments, *n* = 3 replicates each. **d** Oxygen consumption rates (OCR) in 1° adipocytes from indicated depots transfected with scr or *H19* LNA. Alternating backgrounds depict medium, oligomycin, FCCP, and rotenon plus antimycin A injections. Numbers of measured wells are indicated in brackets. **e** OCRs in 1° adipocytes from indicated depots from H19 TG or Controls. **d**, **e** A two-way ANOVA with repeated measurements (2WA-RM) was applied to assess significance. **f**–**h** ORO-stained 6-well plates, ORO photomicrographs and ORO densitometry in **f** BAT, **g** scWAT, and **h** vWAT 1° adipocytes transfected with *H19* (siH19) versus control (siCtrl) siRNAs. An unpaired, two-tailed Student’s *t*-test was applied to assess significance. Scale bar, 250 µm **i** Expression of indicated mRNAs transfected with siH19 or siCtrl. An unpaired, two-tailed Student’s *t*-test was applied to assess significance across *n* = 3–4 experiments, *n* = 3 replicates each. **j** Expression change of 1410 mRNAs differentially expressed (*p* < 0.05, fc ≥ 1.3-fold) in siH19-transfected mature brown adipocytes. **k** Top 5 GO terms enriched (*p* < 0.05, Bonferroni correction) among mRNAs showing significantly higher (top) or lower (bottom) expression (*p* < 0.05) upon siH19 versus siCtrl. **l** GSEA for brown adipogenesis, OxPhos, and mitochondrial biogenesis. *p* empirical *p*-value. **m**, **n** Cumulative distributions of expression changes **(m)** and *p*-values **(n)** for all, BAT-specific, WAT-specific, and common adipogenic genes in siH19-treated mature brown adipocytes. **j**, **m**, **n** Changes are siH19 log2 expression (FPKM) ratios over siCtrl. **o** Proportion of gene sets upregulated (L2R > 0) or downregulated (L2R < 0) in siH19-treated cultured mature brown adipocytes. Unless stated, bar graphs represent mean ± s.e.m. with all data points plotted for (**b**, **c**, **f**–**h**, **i**. *p < 0.05, **p < 0.01, ***p < 0.001. If applicable *p*-values are indicated

When performing locked nucleic acid (LNA)-mediated RNA interference (RNAi) in stromal-vascular fraction (SVF) adipocyte precursor cells isolated from three major adipose depots (BAT, scWAT, and vWAT), we observed blunted lipid accumulation, reduced Oil Red O (ORO) lipid staining, and impaired expression of BAT markers like *Cell Death-Inducing DFFA-Like Effector A (Cidea)*, *Iodothyronine Deiodinase 2 (Dio2)*, and *Ucp1*, as well as reduced UCP1 and PPARG Coactivator 1 Alpha (PGC1A) protein levels, together with common adipose tissue markers like *Adiponectin (Adipoq)*, *CCAAT/Enhancer Binding Protein Alpha (Cebpa)*, *Fatty Acid Binding Protein 4 (Fabp4)*, and *Peroxisome Proliferator Activated Receptor Gamma (Pparg)* upon *H19* RNAi in primary adipocyte precursor cells. We thus concluded that *H19* is required for the commitment of BAT but not scWAT or vWAT-derived progenitors (Fig. 2a–c).

Next, we aimed to dissect the cell-intrinsic and metaboregulatory properties of *H19* using in vitro gain/loss-of-function approaches. Cellular bioenergetics analyses using Seahorse technology confirmed and calculation of coupling efficiencies further supported that *H19* RNAi impaired (Fig. 2d, Supplementary Fig. 1a, c), whilst *H19* overexpression supported oxidative metabolism, uncoupling and extracellular acidification rates (ECAR) in differentiated brown adipocytes (Supplementary Fig. 1b, d, f) and sensitized brown adipocytes to stimulation with beta3-adrenoreceptor agonist CL316,243 (Supplementary Fig. 1e). This illustrates that *H19* supports adipocyte differentiation but also thermogenic gene expression, oxidative metabolism and mitochondrial dynamics in mature adipocytes in a BAT-specific manner. Interestingly, glycolysis was unaltered in *H19* overexpressing adipocytes (Supplementary Fig. 1g), pinpointing towards selective roles of *H19* in mitochondrial lipid oxidation not carbohydrate metabolism.

*H19* was reported to reside in nuclear, but also cytoplasmic and ribosomal fractions in other cell types^11,18^. As LNA-mediated RNAi acts via RNAse H-mediated transcript decay predominantly in nuclei, we next targeted *H19* using small interfering RNAs (siRNAs), which act preferentially in the cytoplasm. We observed *H19* loss across subcellular compartments using both techniques (Supplementary Fig. 1h, i). Doing so, we confirmed critical roles for *H19* in commitment of brown and subcutaneous^19^ adipocytes as demonstrated by decreased ORO staining (Fig. 2f–h) and reduced expression of brown and white adipogenesis markers including *Pgc1a* (Fig. 2i). Expression of adipocyte precursor genes like *Delta Like Non-Canonical Notch Ligand 1* (*Dlk1*), Runt Related Transcription Factor 2 (*Runx2*), *Platelet Derived Growth Factor Receptor Alpha* (*Pdgfra*), *GATA Binding Protein 2* (*Gata2*), *Lymphocyte Antigen 6 Family Member E* (*Ly6a*), and *Transforming Growth Factor Beta 2* (*Tgfb2*) remained high in BAT, but not scWAT or vWAT-derived mature adipocytes upon LNA/siRNA-mediated *H19* silencing (Supplementary Fig. 2a, b). Independent *H19* LNA and siRNA inhibitors evoked similar arrests in brown adipocyte differentiation and impaired expression of BAT markers (Supplementary Fig. 2c, d).

*H19* was historically discovered as monoallelically expressed gene in which solely maternal alleles express *H19* in an epigenetic process termed genomic imprinting. Here, the reciprocally expressed *H19*-*Igf2* imprinted gene cluster critically depends on the enhancer-blocking properties of *CCCTC-Binding Factor (Ctcf)* proteins^20,21^. As no defects in BAT adipogenesis occurred after *Ctcf* RNAi (Supplementary Fig. 2e) and because BAT *Ctcf* and *Igf2* levels were not affected by DIO or cold (Supplementary Fig. 2f, g), we reasoned that cold/DIO-evoked alterations in *H19* are not secondary to altered *H19*-*Igf2* imprinting, corroborating earlier reports about reduced *H19* expression independent from traditional *H19*-*Igf2* imprinting control^22^. Further, the *H19*-encoded microRNA *miR-675* was not expressed in BAT (Supplementary Fig. 2h, i), arguing against the involvement of *miR-675* in brown adipocyte differentiation as observed for myogenic lineage determination^23^.

To gain insights into *H19* function from global expression analysis, we performed RNA-Seq in differentiating siRNA-treated brown adipocytes, and identified 1410 differentially expressed genes (*p* < 0.05, DESeq2), comprising 546 decreased and 999 enriched in siH19 versus siCtrl cells (Fig. 2j). Lower-expressed genes were linked to brown adipocyte and mitochondrial biogenesis and function, which failed to be expressed upon *H19* silencing (Fig. 2j, k top) and Supplementary Data 3), whereas higher-expressed genes were enriched for general functions in signaling, locomotion, and morphogenesis, processes normally suppressed during adipogenesis^24–26^ (Fig. 2j, k bottom and Supplementary Data 3). Gene set enrichment analysis (GSEA)^27^ indicated significant enrichment of gene sets for brown adipogenesis, oxidative phosphorylation (OxPhos), and mitochondrial biogenesis specifically in cells with intact *H19* expression (siCtrl; Fig. 2l). Suppression of the brown adipogenesis program upon *H19* knockdown could be due to suppression of genes important for adipogenesis in general. To test this, we investigated the consequences of *H19* RNAi on expression of previously defined groups of BAT-specific, WAT-specific, and common adipogenic genes in differentiated BAT precursor cells^25^. We found that, in general, loss of *H19* repressed BAT-selective as well as those common adipogenic genes normally activated during adipogenesis (Fig. 2m), which is also reflected by reduced ORO staining (Fig. 2f). In contrast, WAT-selective genes are modestly affected by *H19* knockdown (Fig. 2m, n). Collectively, *H19* mediates the concurrent activation of common adipogenic genes as well as a core BAT gene program, whilst curbing the expression of WAT genes.

### H19 increases BAT EE and prevents obesity

**Fig. 3:**
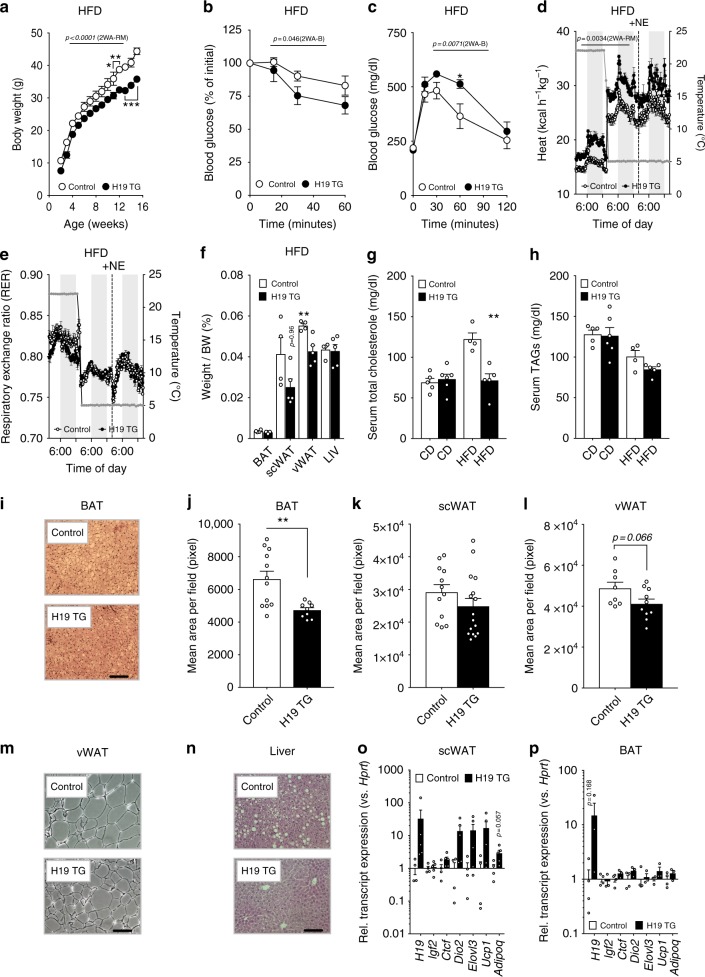
H19 overexpression protects from obesity by increasing energy expenditure and scWAT beiging. **a** Body weight of HFD-fed Control (*n* = 4–5) versus H19 TG (*n* = 4–5) mice. A 2WA-RM with Bonferroni post hoc correction for multiple comparisons (2WA-B) was applied to assess significance. **b** Insulin tolerance test of HFD-fed Control (*n* = 4) versus H19 TG (*n* = 6) mice. **c** Glucose tolerance test of HFD-fed Control (*n* = 5) versus H19 TG (*n* = 5) mice. **d** Energy expenditure in HFD-fed Control (*n* = 4) versus H19 TG (*n* = 5) mice. **b**–**d** A 2WA-B was applied to assess significance. **e** Respiratory Exchange Ratios (RER) in HFD-fed Control (*n* = 4) versus H19 TG (*n* = 5) mice. **f** Tissue/body weight (BW) ratios in HFD-fed Control (*n* = 4) versus H19 TG (*n* = 5) mice. An unpaired, two-tailed Student’s t-test was applied to assess significance. **g** Serum cholesterole levels in HFD-fed Control (*n* = 4) versus H19 TG (*n* = 5) mice. **h** Serum triglyceride levels in HFD-fed Control (*n* = 4) versus H19 TG (*n* = 5) mice. An unpaired, two-tailed Student’s *t*-test was applied to assess significance. **i** Representative photomicrograph of BAT from HFD-fed Control versus H19 TG mice. **j** Automated quantification of adipocyte mean area per field in hematoxylin/eosin (HE) stained BAT sections from HFD-fed Control (*n* = 12 fields) versus H19 TG (*n* = 9 fields). An unpaired, two-tailed Student’s *t*-test was applied to assess significance. **k** Automated quantification of adipocyte mean area in HE-stained scWAT sections from HFD-fed Control (*n* = 12 fields) versus H19 TG (*n* = 16 fields) mice. **l** Automated quantification of adipocyte mean area in HE-stained vWAT sections from HFD-fed Control (*n* = 8 fields) versus H19 TG (*n* = 10 fields) mice. An unpaired, two-tailed Student’s *t*-test was applied to assess significance. **m** Representative photomicrograph of vWAT sections from HFD-fed Control versus H19 TG mice. **n** Representative photomicrograph of liver sections from HFD-fed Control versus H19 TG mice. **m**, **n** Scale bar, 50 µm. **o** scWAT expression of indicated mRNAs from HFD-fed Control (*n* = 4) versus H19 TG (*n* = 5) mice. **p** Relative BAT expression of indicated mRNAs from HFD-fed Control (*n* = 4) versus H19 TG mice (*n* = 3). An unpaired, two-tailed Student’s *t*-test was used to assess significance in **o**, **p**. *p < 0.05, **p < 0.01, ***p < 0.001. If applicable *p*-values are indicated within the panel

Defects in brown fat differentiation and function as well as concomitant impairments in EE render mice susceptible to DIO-induced weight gains and the development of metabolic disease^28–30^. Based on our in vitro findings, we next addressed the role of *H19* in regulating energy metabolism and EE by exposing *H19* gain-of-function^31^ and Controls to HFD or CD. *H19* expression was ubiquitously induced in H19 TG mice without changes in *Igf2* and Ctcf expression or body length alterations (Supplementary Fig. 3a–d). *H19* overexpression strongly prevented DIO-mediated weight gains (Fig. 3a) and improved insulin sensitivity (Fig. 3b) although, interestingly, glucose tolerance was decreased in H19 TG mice (Fig. 3c). The beneficial effect of *H19* overexpression in DIO coincided with increased EE (Fig. 3d), trends towards increased lipid mobilization at 22 °C (Fig. 3e) and oxygen consumption (Supplementary Fig. 3e) at 22 °C, 4 °C and after additional norephinephrine (NE) administration at 4 °C, pinpointing towards improved BAT function in H19 TG mice. This rise in energy catabolism tended towards prevention of DIO-evoked increases in scWAT and vWAT adiposity (Fig. 3f) and precluded DIO-associated elevations in serum cholesterole (Fig. 3g) but not triglycerides (Fig. 3h). *H19* overexpression prevented whitening of BAT (Fig. 3i, j), reduced vWAT but not scWAT hypertrophy (Fig. 3k–m) and development of steatosis (Fig. 3n) in obese animals. The *H19*-dependent induction of EE in obese mice co-occurred with increased expression of browning markers in scWAT at 4 °C, with little alterations in BAT thermogenesis at this temperature (Fig. 3o, p) as reported by others^29^. As *H19* overexpression in lean mice elicited little changes in body weight, insulin sensitivity, RER, glucose clearance, EE and did not affect relative adipose tissue weights and expression of BAT activation markers in lean H19 TG mice (Supplementary Fig. 3f–k), our data suggests that counteracting the HFD-induced *H19* decline is beneficial during DIO, but does not cause detrimental effects in lean mice.

### H19 loss in fat impairs EE and sensitizes towards obesity

**Fig. 4:**
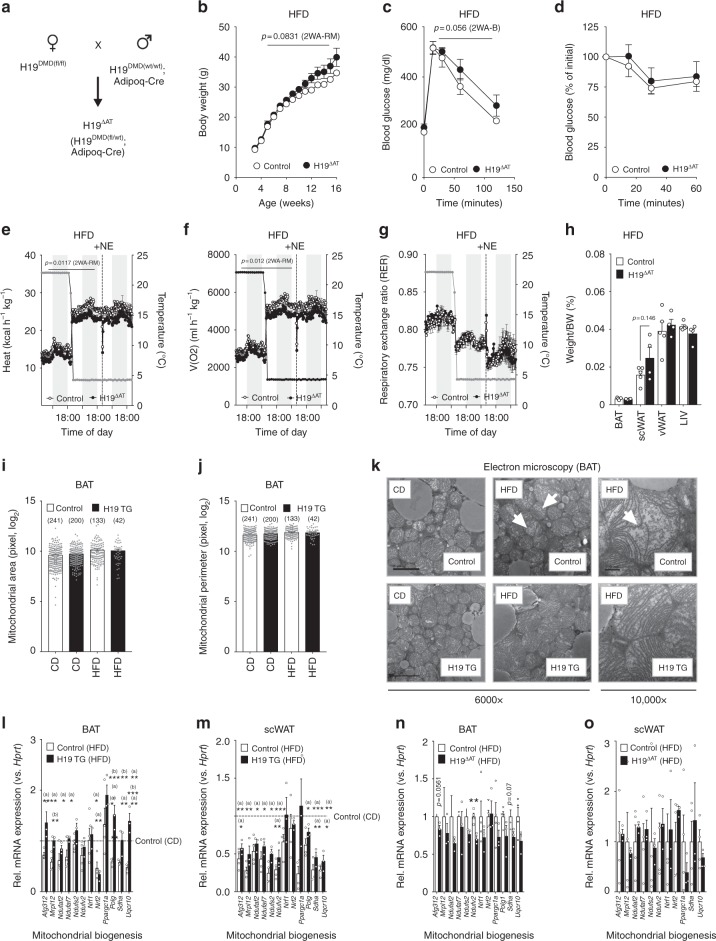
Fat-specific deletion of H19 sensitizes towards DIO-associated weight gains and impairments in energy expenditure. **a** Mating strategy for parental allele-of-origin specific deletion of the *H19* DMR in adipose tissue. **b** Body weight of HFD-fed Control (*n* = 5) versus H19^∆AT^ (*n* = 4) mice. A 2WA-B was applied to assess significance. **c** Glucose tolerance test of male, HFD-fed Control (*n* = 5) versus H19^∆AT^ (*n* = 4) mice. **d** Insulin tolerance test of male, HFD-fed Control (*n* = 5) versus H19^∆AT^ (*n* = 4) mice. **e** Energy expenditure in male, HFD-fed Control (*n* = 5) versus H19^∆AT^ (*n* = 4) mice. **f** Oxygen consumption in male, HFD-fed Control (*n* = 5) versus H19^∆AT^ (*n* = 4) mice. **d**–**f** A 2WA-RM test with repeated measures was applied to assess statistical significance. **g** RER in male, HFD-fed Control (*n* = 5) versus H19^∆AT^ (*n* = 4) mice. **h** Tissue/body weight ratio in male, HFD-fed Control (*n* = 5) versus H19^∆AT^ (*n* = 4) mice. An unpaired, two-tailed Student’s *t*-test was applied to assess significance. **i**, **j** Automated quantification of mitochondrial area (**i**) and perimeters (**j**) in BAT across diets and genotypes (total mitochondria numbers in brackets). An unpaired, two-tailed Student’s *t*-test was applied to assess significance. **k** Representative electron microscopy images from BAT mitochondria across diets and genotypes with magnifications indicated under panel. White arrows depict cristae architectures. Scale bar, 2 µm (6000 × ) or 500 nm (10,000 × ) **l** BAT expression of indicated mRNAs in HFD-fed H19 TG (*n* = 3), HFD-fed Control (*n* = 4), and CD-fed Control (*n* = 5) mice. **m** scWAT expression of indicated mRNAs in HFD-fed H19 TG (*n* = 3), HFD-fed Control (*n* = 4), and CD-fed Control (*n* = 5) mice. **l**, **m** A One-Way ANOVA plus Bonferroni post test was applied to assess statistical significance. ^(a)^ = Significance versus CD-fed Control, ^(b)^ = Significance versus HFD-fed Control. **n** BAT expression of indicated mRNAs in HFD-fed Control (*n* = 4) versus HFD-fed H19^∆AT^ (*n* = 3) mice. **o** scWAT expression of indicated mRNAs in HFD-fed Control (*n* = 4) versus HFD-fed H19^∆AT^ (*n* = 3) mice. An unpaired, two-tailed Student’s *t*-test was applied to assess significance between genotypes. *p < 0.05, **p < 0.01, ***p < 0.001. If applicable *p*-values are indicated within the panel

As H19 TG mice ubiquitously overexpress *H19*, we next ablated *H19* specifically in BAT by crossing female mice harboring loxP-flanked *H19* methylation-sensitive imprinting control regions (ICRs) with male *Adipoq*-cre expressing animals (H19^∆AT^, Fig. 4a, Supplementary Fig. 4a, b). H19^∆AT^ knockout mice gained more body weight compared to Controls upon HFD (Fig. 4b) but not CD (Supplementary Fig. 4c) feeding with negligible alterations in insulin sensitivity or glucose tolerance (Fig. 4c, d and Supplementary Fig. 4d, e). Crucially, BAT-specific *H19* loss decreased EE and oxygen consumption in both diets (Fig. 4e, f and Supplementary Fig. 4f, g), tended towards decreased lipid oxidation at 4 °C and increased scWAT adiposity in obese (Fig. 4g, h), yet not lean (Supplementary Fig. 4h, i) mutant mice, suggesting decreased BAT function in H19^∆AT^ mice, particularly during DIO. As primary brown adipocytes isolated from H19^∆AT^ mice exhibited defects in mature brown adipocyte function (Supplementary Fig. 4j, k) as partly also observed during *H19* RNAi (Supplementary Fig. 4p), we reasoned that impairments in terminal brown function contribute to the observed defects in EE and susceptibilities towards DIO in H19^∆AT^ mice.

### Fat hH19 is reduced in obese humans

As *H19* is strongly conserved^32^ and human *H19* (*hH19*) is expressed in mature adipocytes compared to non-parenchymal cells in fat (Supplementary Data 4), we next asked if *hH19* could be affected by obesity also in humans and quantified *hH19* levels in scWAT and vWAT biopsies from 169 lean and obese patients. We observed that *hH19* expression declined with ascending BMIs in both depots and correlated positively with markers of adipose beiging like *UCP1* mRNA levels (Supplementary data 5), underscoring that *hH19* could support energy dissipation and acquisition of catabolic expression profiles in human fat.

### H19 regulates brown adipose mitochondrial biogenesis

Cold temperature activation of BAT thermogenesis and the concurrent turnover of nutrients, such as carbohydrates and lipids requires active mitochondrial biogenesis and rearrangements of mitochondrial meshworks via fission and fusion^33,34^. Based on the cell-intrinsic alterations in oxidative metabolism observed in *H19*-deficient and overexpressing adipocytes (Fig. 2d, e, Supplementary Fig. 1a, b) we studied mitochondrial morphologies using electron microscopy. Although gross parameters like mitochondrial area or perimeter (Fig. 4i, j) were unchanged, we observed substantial defects in mitochondrial architecture such as perturbed cristae formation in obese Control but not H19 TG mice (Fig. 4k), while H19^∆AT^ mitochondria showed impaired cristae upon HFD feeding (Supplementary Fig. 4q). The expression of genes involved in mitochondrial biogenesis was blunted in obese BAT, whilst *H19* overexpression reversed these DIO-evoked defects in gene expression in BAT, but not scWAT (Fig. 4l, m). H19^∆AT^ mice displayed less pronounced reductions of the same gene set in BAT (Fig. 4n) yet not scWAT (Fig. 4o). In contrast, fission and fusion parameters were unaltered in BAT and scWAT (Supplementary Fig. 4l–o). Interestingly, cell-intrinsic defects in mitochondriogenesis occurred also in *H19*-deficient brown adipocytes (Supplementary Fig. 4p), closely mimicking those elicited by DIO or *H19* loss in adipose tissue itself. Collectively, we here show that *H19* loss in adipose tissue renders mice susceptible to DIO weight gains and impairments of EE, potentially due to the control of mitochondrial dynamics by the lincRNA *H19*.

### BAT represents a unique case of tissue-specific imprinting

**Fig. 5:**
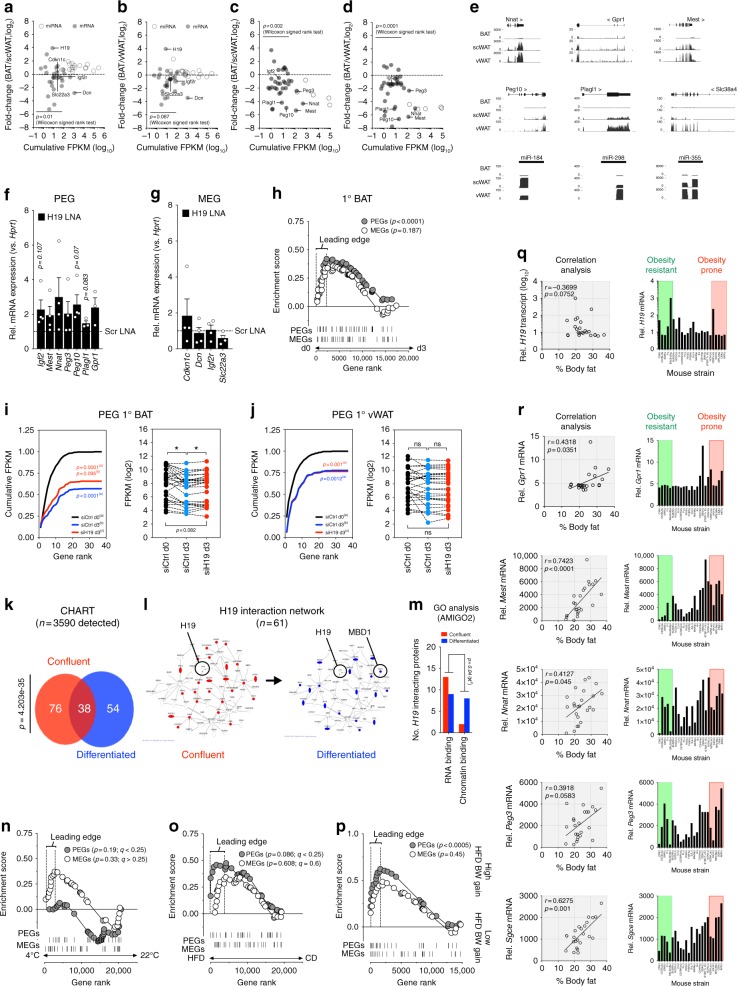
Repression of BAT paternal monoallelic gene expression by the lincRNA H19. **a** Plot of expression fold-changes of maternally expressed genes (MEGs) in BAT versus **a** scWAT **b** vWAT. **c** Plot of expression fold-changes of paternally expressed genes (PEGs) between BAT versus **c** scWAT **d** vWAT. A Wilcoxon matched-pairs signed rank test was used to assess statistical significance for up- or downregulation of PEGs/MEGs (**e**) UCSC Genome Browser showing PEG abundances in BAT, scWAT, and vWAT. **f**, **g** Expression of indicated PEGs (**f**) or MEGS (**g**) in BAT 1° adipocytes transfected with scr or *H19* LNA. **f**, **g** A paired, two-tailed Student’s *t*-test was used to assess significance across *n* = 4 experiments, *n* = 3 technical replicates each. **h** GSEA showing downregulation of PEGs but not MEGs (list from www.geneimprint.com) during 1°BAT differentiation. **i**, **j** Cumulative distribution frequency (left) and abundances (right) of PEGs in **i** 1°BAT and **j** 1°vWAT in siCtrl transfected adipocyte progenitors at d0 (black) versus differentiated siCtrl (blue) and siH19-transfected (red) 1° adipocytes at d3. **k** Quantification and overlap of *H19* co-immunoprecipitating proteins in confluent (red) and differentiated (blue) PIBA cells determined by CHART-MS. Pulldown of *H19*-interacting proteins was performed using six *H19*-specific antisense/sense oligonucleotides in *n* = 3 replicates. **l** Illustration of *H19* interaction network comprising *n* = 61 proteins generated using Ingenuity Pathway Analysis. Red and blue nodes depict proteins co-immunoprecipitating in confluent and differentiated cells, respectively. **m** AMIGO2 GO classification of *H19* interactors across cellular states. **n**–**p** GSEA analysis of PEG gene ranks in mice **n** exposed to 4 °C cold stress for 24 h, **o** HFD feeding, or **p** exhibiting different susceptibility to DIO-evoked weight gains^53^. **q**, **r** Correlation of WAT (**q**) *H19* or (**r**) PEG abundances versus a ranked list of 24 obesity-prone and –resistant non-isogenic mouse strains. *H19*/PEG abundances were from (MOE430 V2)—Adipose (www.biogps.org54) and body composition after HFD feeding defined as obesity-resistant versus obesity-prone strains^65^. Significance of association between expression versus (%) body fat was determined using Spearman’s correlation analysis. *p < 0.05, **p < 0.01, ***p < 0.001. If applicable additional p-values are indicated within the panel

The lincRNA *H19* represents a quintessential representative of a class of imprinted genes exclusively transcribed from one parental allele^20,35^. Although monoallelic expression is only observed in <1% of all genes in eutherians^36^, loss-of-function (epi)-mutations in individual imprints or imprinted gene clusters cause severe imprinting disorders (ID) like Prader–Willi’s, Beckwith–Wiedemann’s, and Angelman’s syndromes^37^, in Silver–Russell’s syndrome even due to defects in the *H19*-*Igf2* locus^38^. Despite progress in understanding the molecular underpinnings of imprinting, the etiology of IDs^21,39,40^ and even recent approaches to correct IDs^41^, the question remains why certain genes but not others are monoallelically expressed per se^42^ and why certain genes are expressed from one parental gender, not the other. Thus, to date it remains unsolved whether functional or conceptual similarities between paternally expressed (PEGs) or maternally expressed genes (MEGs) as two discordant gene sets exist at all, largely because PEGs/MEGs represent a collection of genes with heterogeneous functions. Interestingly, one hallmark of ID patients and ID mouse models are metabolic problems, for instance altered adiposity and lipodystrophy and alterations in insulin sensitivity and EE^43^, all processes closely linked to brown and WAT (dys)-function. Given the BAT-selectivity of *H19* and its novel role in brown adipocyte differentiation and function, we were interested how other PEGs/MEGs are expressed across fat depots transcriptome-wide. While RNA-Seq from C57BL/6 mice revealed no gross differences in MEGs abundances between BAT, scWAT, or vWAT, seven fat-abundant protein-coding (*G Protein Coupled Receptor 1 (Gpr1)*, *Insulin Like Growth factor 2* (*Igf2*)*, Mesoderm Specific Transcript (Mest), Neuronatin (Nnat)*, *Paternally Expressed 3 and 10 (Peg3, Peg10)*, and *Plag1 Like Zinc Finger 1 (Plagl1)*) and three microRNA PEGs (*miR184, miR298*, and *miR-335*, Fig. 5e) and many others were absent from BAT yet robustly expressed in scWAT and vWAT (Fig. 5a–e).

### H19 curbs expression of PEG-enriched gene networks in BAT

To shed molecular light on this unique pattern of fat tissue-specific imprinting, we built upon recent reports demonstrating that expression of imprinted genes can be interdependent, e.g., during muscle regeneration or in embryonic fibroblasts^44–47^ and that PEG-enriched imprinted gene networks (IGNs) are affected by *H19*^48–50^. We thus hypothesized that *H19* represses PEG-IGNs in brown fat and, indeed, *H19* RNAi tended to derepress fat-abundant PEGs like *Igf2, Peg10*, and *Plagl1* but not MEGs like *Cyclin Dependent Kinsae Inhibitor 1C (Cdkn1c), Decorin (Dcn), Igf2 Receptor (Igf2r)*, and *Solute Carrier Family 22 Member 3 (Slc22a3)* (Fig. 5f, g). As PEG expression declined brown adipogenesis (Fig. 5h), we next asked if *H19* ablation affects PEG/MEGs globally. As expected, *H19* loss did not alter MEG expression in differentiated primary (1°) BAT or 1°vWAT precursor cells (Supplementary Fig. 5a, b), yet counteracted differentiation-induced PEG losses during brown, but not white adipogenesis (Fig. 5i, j). Thus, *H19* acts as PEG gatekeeper in brown adipocytes, a finding not observed for BAT MEGs nor white fat PEGs.

### H19 represses BAT PEGs by recruiting MBD1 chromatin modifier

As *H19* expression was not altered in BAT, scWAT, and vWAT commitment (Supplementary Fig. 5c) and because we did not observe differences in DNA methylation of affected PEGs ICRs (Supplementary Fig. 5d), we asked whether specific protein interactors of *H19* control its PEG gatekeeper function selectively in brown adipocytes. We thus conducted Capture Hybridization Analysis of RNA Targets^51^ coupled to mass spectrometry (CHART-MS) from confluent and differentiated primary immortalized brown adipocytes (PIBA) and obtained a comprehensive *H19* protein interactome from both conditions: In total 3590 proteins were detected by MS, 168 were enriched by *H19* antisense, not sense oligonucleotide co-immunoprecipitation (Supplementary Data 7). 76 and 54 proteins bound to *H19* in confluent and differentiated PIBA cells, respectively, and 38 proteins were bound by *H19* in both (Fig. 5k). Next, we performed Ingenuity Pathway Analysis to construct an interconnected *H19* binding protein network comprising 61 proteins (Fig. 5l). Crucially, when performing gene ontology (GO) analyses, we observed that whereas in immature (confluent) brown adipocytes *H19* mostly recruited proteins annotated as RNA-binding proteins (RBPs, e.g., *H19* interactors like *Insulin Like Growth Factor 2 Binding Protein 3 (IGFBP3)*)^52^, in differentiated cells *H19* preferentially associated with proteins classified as chromatin binders or chromatin modifiers (Fig. 5l, m). Of note, *H19* bound DNA methyltransferase MBD1 (and its homolog MECP2) only in differentiated PIBA cells. Crucially, *H19*-MBD1 complexes affect histone 3 lysine methyltransferases (KMT) dependent deposition of repressive H3K27 trimethylation marks and *H19* or MBD1 loss both resulted in reduced H3K9me3 at PEG loci like *Igf2*, *Solute Carrier Family 38 Member 4 (Slc38a4)* and *Mest/Paternally Expressed 1 (Peg1)*^50^. Accordingly, we observed in primary mature brown adipocytes that siH19 and/or siMbd1 RNAi increased BAT-abundant PEGs with similar tendencies, arguing for functional H19/MBD1 complexes in these cells (Supplementary Fig. 5e). Presence of *H19*-MBD1 complexes occurring solely in mature brown adipocytes thus supports the notion of *H19* as PEG gatekeeper in brown adipocytes, potentially via MBD1-dependent alterations of H3K9me3 KMTs recruitment. Crucially, the same phenomenon does not occur in white adipocytes where *H19* abundances are low.

We reasoned that PEG enrichment in subcutaneous and visceral WAT pinpoints towards an underappreciated involvement of PEGs in WAT-relevant processes like white adipogenesis or lipid accrual, i.e.,processes that sensitize towards increased adiposity and obesity. To address this, we quantified PEGs/MEGs in DIO using RNA-Seq and, performing GSEA^27^, observed trends towards decreased PEG levels in cold-exposed (Fig. 5n) and elevated PEG expression in obese BAT (Fig. 5o). In an elegant study, Koza et al. biopsied adipose tissue from C57BL6 mice before HFD feeding^53^. Profiling the pretreatment samples from low and high BW gainers of the 107-animal cohort, the authors reported gene signatures for DIO susceptibility. Reanalyzing this data set, we found clear evidence that PEG levels foreshadow obesity susceptibility even before overnutrition (Fig. 5p). Finally, *H19* correlated negatively, whereas 5 of 10 fat-abundant PEGs (*Gpr1, Mest*, *Nnat*, *Peg3*, and *Sgce*) correlated positively with DIO susceptibility across a panel of 25 phenotypically and genetically discordant mouse strains^54^ (Fig. 5p–r, Supplementary Fig. 5f). Taken together, we believe that *H19*-MBD1 complexes repress PEG but not MEG expression in brown adipocytes, supporting a notion according to which individual PEGs^47,55,56^ but also paternal monoallelic gene expression in general sensitizes towards fat accumulation and diet-induced weight gains, a process at least partially repressed by the lincRNA *H19* in BAT.

Collectively, we here demonstrate that the conserved lincRNA *H19* is uniquely required for differentiation and mature fat cell function of brown but not white adipocytes in vitro and that brown adipose *H19* ensures energy dissipation in vivo. One mechanism of how *H19* supports EE could be its cell-intrinsic control of mitochondrial biogenesis in mature brown adipocytes and the ensurance of maintaining quiescence of obesity-predisposing PEGs in BAT.

## Discussion

Our results collectively indicate that the conserved lincRNA *H19* promotes brown adipocyte differentiation and function in vitro and that *H19* ensures energy dissipation in vivo. In addition, we observed that *H19*-mediated alterations of IGN constitutes an important regulatory layer of BAT development and function according to which PEGs affect brown fat negatively (*Dlk1*, *Peg1*, *Ndn*, paternally expressed GNAS isoform XLαs), whereas MEGs (Gαs) affect BAT positively^43^. The kinship theory of the evolution of imprinting predicts that PEGs should act to reduce energy-costly thermogenic output^57^. Our data are broadly consistent with this prediction as loss of *H19* in brown adipocytes leads to coordinated transcriptional upregulation of multiple paternally imprinted genes. This finding is distinct from canonical imprinting regulation in that perturbed imprinting control should lead to downregulation of some imprinted genes with a reciprocal upregulation of others.

We obtained no evidence for changes in canonical imprinting in our models, amongst others because DNA methylation of affected PEGs ICRs and *Igf2*/*Ctcf* expression was unaltered by *H19* loss in brown adipocytes. Our data rather suggest that this unique pattern of PEG regulation is maintained through *H19*/Mbd1 complexes acting in *trans*. Although evidence supporting this was collected from a single *Mbd1* siRNA, MBD1 was already shown to interact with *H19* to regulate IGNs during embryo growth^50^. Yet, future studies are needed to show if *H19* PEG gatekeeper function persists at thermoneutrality or other (e.g., surgical) models of altered BAT-evoked EEs.

Because of the role of PEGs in WAT-relevant processes like white adipogenesis and adipocyte cell size regulation, it is plausible to assume that *H19* preserves BAT homeostasis during DIO by its cell-intrinsic control of mitochondrial biogenesis in mature brown adipocytes and by maintaining quiescence of obesity-predisposing PEGs in BAT. In line with this hypothesis are data showing brown adipocyte death as a consequence of DIO induce whitening of BAT^58^, a process suppressed by *H19*.

Collectively, our data suggest that *H19* acts as PEG gatekeeper in brown adipocytes, potentially acting via MBD1-dependent alterations of H3K9me3 KMTs recruitment are in line with previous reports demonstating that *H19* regulates fine-tuned regulation of embryonic growth mediated by the *H19* gene. Identification of the underlying molecular mechanisms through which it controls its targets is an important issue.

## Methods

### Animal care and research diets

All animals were maintained on a C57BL/6 background, housed in groups of 3–5 animals per cage on a constant 12 h light/dark cycle in a SPF-controlled facility with regular testing for pathogens. All experimental mice were 17–18 weeks of age at sacrifice. Experimental mice were exposed to HFD feeding for 15 weeks (H19 TG and H19^∆AT^ mice versus littermate Controls) and 17–18 weeks of age at sacrifice. Care of animals was within institutional and animal-care committee guidelines approved by local (Bezirksregierung Köln) or regional (Tierschutzkommission acc. §15 TSchG of the Landesamt for Natur, Umwelt und Verbraucherschutz (LANUV) North-Rhine Westphalia, Germany, internal accession no. 84–02.04–2014.A068) authorities. Unless otherwise indicated, animals were allowed ad libitum access to control diet (CD, D12450B* mod LS; Sniff) containing 62 kJ% carbohydrates, 27 kJ% protein, and 11 kJ% fat and drinking water. DIO was achieved by feeding a high-fat diet (HFD, D12492 (I) mod; Sniff) containing 22 kJ% carbohydrates, 24k J% protein, and 54 kJ% fat from starting at 3 weeks of age.

### Mouse husbandry

Adipose tissue samples used for acquisition of a tissue RNA-Seq profile were taken from male C57BL/6 mice (Charles River). At time of sacrifice, the mice were 32 weeks of age and 28 weeks exposed to CD feeding. C57BL/6 male mice used for CD versus HFD feeding came from Charles River. Upon dissection, the mice were 32 weeks of age and 28 weeks exposed to CD or HFD feeding. For cold exposures, 10–12 weeks old C57BL/6 male mice were either constantly housed at 22 °C or exposed to 4 °C cold challenges for 20 h, fasted for additional 4 h, and sacrificed thereafter. *H19* overexpression was achieved using a mouse strain harboring yeast artificial chromosomes (YACs, present in ten genomic copies) with each YAC cassette containing a 130 kb transgene spanning the entire *H19*-Igf2 cluster with a LacZ-inactivated *Igf2* coding sequence (MGI accession number 5648556, Symbol: Tg(Igf2/LacZ,H19)YZ15Aco; H19 TG)^31^. Non-transgenic littermates were used as controls. Only male, non-randomized mice were used, the investigator not blinded to genotypes due to strong phenotypical differences (e.g., in body weight upon HFD feeding) between H19 TG and Controls. Mice harboring loxP-flanked *H19* differentially methylated regions (DMR; H19-DMR^flDMR/flDMR^) were generated as described^59^ and obtained from Linheng Li (Stowers Institute). Female H19-DMR^flDMR/flDMR^ were bred with male C57BL/6 expressing adipose-selective Adiponectin (*Adipoq*)-promoter driven Cre (H19-DMR^wt/wt^; Adipoq-Cre^tg/wt^) to obtain H19-DMR^flDMR/wt^; Adipoq-Cre^tg/wt^ (H19^∆AT^) and H19-DMR^flDMR/wt^; and Adipoq-Cre^wt/wt^ control littermates (Control to H19^∆AT^). Only male, non-randomized mice were used with the experimentator not blinded to the genotype.

### Mouse RNA isolation

RNA from indicated tissues and primary adipocytes was isolated using Trizol® according to manufacturer’s protocols for total RNA isolation. For subcellular localization analyses, RNAs were purified from nuclear and cytoplasmic fractions obtained using the PARIS kit (Ambion) according to manufacturer’s protocol.

### Deep RNA-sequencing procedure

(1) For 4 °C versus 22 °C and CD versus HFD BAT samples: library preparation and sequencing was performed at the Max Planck-Genome-centre Cologne, Germany. Following initial quality checks, 1 µg total RNA of each sample was depleted for rRNA using NEBNext® rRNA depletion Kit (human/mouse/rat). Library preparation was performed with NEBNext Ultra™ Directional RNA Library Prep Kit for Illumina (New England Biolabs). All libraries were sequenced in parallel on a HiSeq2500 instrument (Illumina) in 2 × 100 bp sequencing mode. (2) For siH19 and siCtrl transfected 1° BAT and 1° vWAT samples: library preparation and sequencing was carried out by the Core facility Genomics, Medical University of Vienna, Vienna, Austria. Briefly, sequencing libraries were prepared using the NEBNext® Ultra™ II RNA Library Prep Kit according to manufacturer’s instructions and sequenced on Illumina NextSeq550 platforms. The resulting 75 bp single-end reads were quality-checked with FastQC (http://www.bioinformatics.babraham.ac.uk/projects/fastqc/), and low quality reads were removed using the fastq_quality_trimmer (“-t 20 -l 25” parameters) from the FASTX-Toolkit.

### Deep RNA-sequencing data processing

RNA-Seq data were processed using the QuickNGS analysis pipeline^60^, version 1.2.7, based on Ensembl release 87. In brief, reads were mapped to the GRCm38 assembly of the mouse genome using Tophat2, version 2.0.10, and reassembled with Cufflinks, version 2.1.1. Differential gene expression was analyzed using the DESeq2 package, version 1.10.1. The results were uploaded to the QuickNGS database and combined with multiple annotations using the biomaRt package. For 4 °C versus 22 °C and CD versus HFD BAT samples, *n* = 2 per condition and for siH19 and siCtrl transfected 1° BAT and 1° vWAT samples, *n* = 3 per condition.

### Gene set enrichment analysis

GSEA is a computational pathway analysis tool that determines if a given set of manually curated genes show statistically significant, concordant differences between two biological states (http://www.broadinstitute.org/gsea/index.jsp). A list of murine imprinted genes from www.geneimprint.com was used to construct gene sets representing all known PEGs and MEGs, respectively. Genes were first ranked based on real value using the weighted signal-to noise metric. *p*-values and false discovery rates (FDR) for the enrichment score of each gene set were calculated based on 1000 gene set permutations.

### Cultivation of PIBA cell lines

PIBA cell lines were generated in-house (s. below) and maintained in growth medium Dulbecco’s modified Eagle’s medium (DMEM, PAA) containing 4.5 g/l glucose supplemented with 10% FCS, 5 mM L-glutamine, 0.1 mM non-essential amino acids, 1 mM sodium pyruvate and 1× penicillin/streptomycin (P/S). Cells were grown to confluency in differentiation medium (DMEM, 20 nM insulin, 1 nM T3). Brown adipocyte differentiation was induced using differentiation medium supplemented with 0.125 mM indomethacin, 2 mg/1 dexamethasone, and 0.5 mM isobutylmethylxanthine for 1 day and incubated in differentiation medium for 5 more days.

### Generation of stable PIBA cell lines

PIBA cells were prepared from C57BL/6 mice based on principles described in Klein et al.^61^ with slight modifications. Interscapular BAT from postnatal day three newborn-mice were resected and minced in 500 µl sterile PBS. Tissue pieces were subjected to collagenase digestion by adding 500 µl digestion buffer (123 mM NaCl, 5 mM KCl, 1.3 mM CaCl2, 5 mM glucose, 100 mM HEPES, 1% P/S, 4% BSA) containing 1.5 mg/mL Collagenase A (Roche), followed by repeated cycles of vortexing for 10 s and incubation at 37 °C for 30 s every 5 min until a single cell suspension was achieved. Cell suspensions were filtered through a 100 µm screen and cells collected by centrifugation at 422.2× *g* for 5 min. The cells were then resuspended in Dulbecco’s modified Eagle’s medium (DMEM) supplemented with 4.5 g/l glucose, 20% fetal calf serum (FCS), 20 mM HEPES, sodium pyruvate, L-glutamine, non-essential amino acids and 1% P/S and cultured at 37 °C with 5% CO2. Media was changed every day until cells had reached 80% confluency and were passaged once. For immortalization cells were transfected with a plasmid encoding the SV40 large T-antigen using Lipofectamine 2000 transfection-reagent (Invitrogen) according to the manufacturer’s instructions. PIBA cells were tested mycoplasma negative before experiments.

### Isolation stromal-vascular fraction derived 1° adipocytes

To isolate depot-specific SVF cells, male C57BL/6 mice were sacrificed by CO2 or cervical dislocation. Next, interscapular BAT, posterior inguinal subcutanous (scWAT) and perigonadal visceral (vWAT) tissue of 6–8 weeks old male of indicated genotypes was removed and tissues transferred into serum-free DMEM/Ham’s F-12 medium. To obtain sufficient cell numbers, BAT tissues were pooled according to genotype if needed. In a sterile environment, tissue medium was aspirated before cutting the adipose tissue into pieces until a homogenized consistency was achieved. Homogenized tissue was collected in a 50 ml tube filled with 10 ml serum-free DMEM/Ham’s F-12 medium. To wash out cellular debris, 5 ml of the lower phase without visible tissue chungs were aspirated, 5 ml serum-free DMEM/Ham’s F-12 medium added and in a final wash 5 ml of the infranatant removed. For each scWAT and vWAT tube 1 mg/ml Collagenase II, 1. 5% BSA, 6.6 ml serum-free medium and 100 µl DNase (15 kU/ml) were prepared. For each BAT tube the same substances as for scWAT and vWAT plus 240 µl Dispase I (50 U/ml) were added. To each adipose tissue preparation, 10 ml of the mixed enzyme solution were added. Next the tubes were placed in a 37 °C water bath and shaken at 120 rpm/min for 15 min. The tubes were vigorously shaken by hand before warming again at 37 °C for another 15 min at 120 rpm/min. For purification, the cell suspension was filtered through 100 µm filters into a 50 ml tube filled with 20 ml DMEM/Ham’s F-12 medium. The suspension was centrifuged for 5 min, 21 °C and 200× *g*, supernatants discarded and pellets resuspended in 1 ml DMEM/Ham’s F-12 growth medium (DMEM/Ham’s F12 medium plus 10% Fetal Calf Serum, 1% P/S, 0.1% Biotin and 0.1% pantothenic acid) and filled up to 10 ml. After centrifugation for 5 min, 21 °C and 200× *g*, the medium was aspirated up to 1 ml, the pellet resuspended and transferred into a 15 ml tube. The tube was filled up to 13 ml with growth medium and centrifuged for 5 min, 21 °C and 200× *g*. Next, supernatants were aspirated and pellets resuspended in 1 ml Erythrocyte Lysis Buffer for 5 min. The tube was filled up to 13 ml and centrifuged for 5 min, 21 °C and 200× *g*. The supernatant was aspirated up to 1 ml solution. In the remaining 1 ml, the pellet was resuspended and the solution filtered using a 30-µm filter wetted with 500 µl growth medium. After filtration, the filter was rinsed with 500 µl growth medium and isolated cells counted using a Neubauer Counting Chamber to seed 50,000 cells/well in flat bottom 24-well-plates.

### Induction of SVF adipogenesis

To induce commitment of SVFs into mature adipocytes, freshly prepared 0.05% Insulin, 0.005% Dexmethasone, 0.001% Rosiglitazone, and 0.05% 3-Isobutyl-1-methylxanthin (IBMX, scWCAT and vWAT) or 0.1% Indomethazine, 0.001% Triiodothyronine (BAT) in growth medium (induction medium) were added to confluent cells. After 48 h of induction, differentiation was initiated using freshly prepared 0.001% Rosiglitazone (scWAT and vWAT) or 0.001% Triiodothyronine (BAT) in growth medium (differentiation medium). Differentiation was achieved after 3–4 days of incubation in differentiation medium.

### LNA-mediated gene knockdown of primary adipocytes

Primary brown adipocytes were derived from depots-specific SVFs as described above. Cells were grown until confluency in growth medium. For *H19* inhibition custom-made LNA GapmeRs were transfected after cells reached confluency (GapmeR sequences are provided in Supplementary Data 6). Lipofectamine 2000 was diluted 1:62.5 in Opti-MEM. For a final concentration of 25 nM, respective LNAs (stock 10 µM) were diluted 1:50 in Opti-MEM. Both solutions were incubated for 5 min at RT. LNA and Lipofectamine solutions were mixed at equal volumes and incubated for 20 min at RT. The cells were washed with pre-warmed PBS and 750 µl of growth medium without P/S added to each well. A volume of 250 µl of LNA/Lipofectamine mix was added and cells incubated for 24 h at 37 °C and 5% CO_2_ before changing medium to induction medium.

### siRNA-mediated gene knockdown of primary adipocytes

SiRNAs targeting *H19* (siH19, oligo n253571) or non-targeting Control (siCtrl, Cat. No. 4390843; both 100 nmol/l, Invitrogen) were delivered into 1° BAT or vWAT adipocytes by Amaxa nucleofection (Lonza Bioscience) according to manufacturer’s recommendations. The cells were utilized 48–72 h after transfection. SiRNA sequences are provided in Supplementary Data 6.

### Immunoblot analysis

For protein isolation, the medium of cells was aspirated and the plates were stored on ice. The cell monolayer was washed gently one time with ice-cold PBS. Excess PBS was aspirated. A volume of 50 µl of RIPA lysis buffer with inhibitors was added to each well of the 24-well plate. RIPA buffer was composed of 50 mM Tris-HCL pH 7.5, 150 mM NaCL 1 mM EDTA, 0.1% sodium deoxycholate, 1% NP-40, 1× protease inhibitor and 1× phosphatase inhibitor. An ice-cold cell scraper was used to scrape the cells. The lysate was transferred to 1.5 ml Eppendorf tubes. The samples were snap-frozen in liquid nitrogen and thawed on ice for three repeated cycles. After 10 min of centrifugation at 12,000× *g* and 4 °C the supernatant was transferred to fresh tubes and stored on −80 °C. Protein concentration was determined using the bicinchonoinic acid method. Samples were separated by SDS-PAGE after being mixed with 4× Laemmli Sample Buffer containing 10% β-mercaptoethanol and heated to 95 °C for 5 min. Afterwards, proteins were transferred to nitrocellulose membrane for incubation with primary antibodies raised against PGC-1A (sc-13067, Santa Cruz 1:500), UCP1 (sc-6528, Santa Cruz, 1:500). Calnexin (208–880, Calbiochem, 1:5000) served as loading control. Uncropped scans of the blots can be found as a Supplementary Figure in the Supplementary Information.

### Oil Red O Staining

ORO solution was prepared by dissolving 0.3 g ORO dye in 60 ml isopropanol in the dark overnight at room temperature. Afterwards, 40 ml dH2O was added and the solution filtered. In a fume hood the media of the cell plates was aspirated and plates rinsed with 2 ml of sterile PBS per well. Next, PBS was aspirated and 1 ml of 10% formalin added. Cells were incubated for 1.5 h at room temperature, excess formalin removed, wells washed with 2 ml PBS and stained with 1 ml ORO staining solution for 2 h. Before image acquisition, the wells were washed twice with dH2O for 5 min.

### Oxygen consumption rates and glycolytic activity

SVF from indicated adipose tissue depots were seeded into Agilent Seahorse XFe96 Bioanalyzer microplates. Per well 50,000 cells were seeded and incubated in DMEM/Ham’s F-12 medium plus 10 % Fetal Calf Serum, 1% P/S, 0.1% Biotin and 0.1% Pantothenic acid (Growth medium) at 37 °C and 5% CO2 at a standard incubator until confluency is reached. For *H19* inhibition cells were transfected as described above directly in the Seahorse setup. For this, the cells were washed with pre-warmed PBS and 75 µl of growth medium w/o P/S added to each well. A volume of 25 µl of LNA/Lipofectamine mix was added and cells incubated for 24 h at 37 °C and 5% CO_2_ before changing medium to induction medium. To induce commitment of SVFs into mature adipocytes directly within the Seahorse microplates, freshly prepared 0.05% Insulin, 0.005% Dexmethasone, 0.001% Rosiglitazone, and 0.05 % IBMX (scWCAT and vWAT) or 0.1% Indomethazine, 0.001% Triiodothyronine (BAT) in growth medium (induction medium) were added. After 48 h of induction, differentiation was initiated using freshly prepared 0.001% Rosiglitazone (scWAT and vVAT) or 0.001% Triiodothyronine (BAT) in growth medium (differentiation medium). Differentiation was achieved after 3–4 days of incubation in differentiation medium. For each seahorse plate the corresponding calibration plate was prepared 24 h prior to experiments using 200 µl XF Seahorse Calibrant Agilent per well. The plate was incubated for 24 h in a non-CO2 incubator at 37 °C and the instrument set to 37 °C 24 h prior to the experiment. One hour before measuring the plate, it was washed with PBS and the medium changed according to the corresponding experiment analyte kits (MitoStressKit or GlycoStressKit, provided by the manufacturer). Prior to measurement, calibration was started using calibration plates, measuring O2 and pH LED Value/emission/Initial reference Delta for each well. After calibration cartridges were kept within the machine and measurement of adipocyte-containing microplates commenced. Measurement parameters were: Mix: 3 min, wait 0 min, measure 3 min with each reagent’s effect assessed within 3 (MitoStressKit) or 4 measurement cycles (GlycoStressKit) with a total duration of 18 or 24 min per reagent injection. All measurements started with measuring basal values, followed by injection of Oligomycin, FCCP and Rotenone + Antimycin A (MitoStressKit) or Glucose, Oligomycin and 2-Deoxy-Glucose, GlycoStressKit). Coupling efficiencies were calculated as reported recently^62^.

For MitoStress Kits, corresponding media were prepared shortly before the experiment and consisted of Basal Seahorse Medium supplemented with 25 mM Glucose, 1 mM Glutamine, 2 mM Sodium Pyruvate, set to pH = 7.4 and filtered sterile. Per Plate ca. 25 ml of MitoStress Medium were needed and Seahorse cell plates were changed to 180 µl MitoStress medium 1 h prior to calibration in a non-CO2 incubator at 37 °C. The calibration plate possessed a cartridge having 4 pockets per well. Before the measurement pocket A was filled with 20 µl 10 µM Oligomycin, pocket B with 22 µl 10 µM FCCP and pocket C with 25 µl 5 µM Antimycin A and Rotenone. For the GlycoStressKit, the cell plate was washed with PBS and media changed to filtered 180 µl GlycoStress medium. GlycoStress medium consisted of Basal Seahorse Medium supplemented with 1 mM Glutamine and 2 mM Sodium Pyruvate, set to pH = 7.4 and stored for 1 h in a non-CO2 incubator at 37 °C. The calibration plate possessed a cartridge having 4 pockets per well. Shortly before the measurement pocket A was filled with 20 µl 10 mM Glucose, pocket B with 22 µl 10 µM Oligomycin and pocket C with 25 µl 50 mM 2-Deoxy-Glucose.

### Glucose tolerance test & insulin tolerance test

At the time of performing insulin tolerance test (ITTs), mice were 12 weeks of age and 9 weeks exposed to CD or HFD feeding. The ITT was carried out in random-fed mice at 9–10 am in the morning in fresh cages with bedding, free access to drinking water but no food. After determining basal blood glucose levels (0 min), each animal received 0.75 U/kg of body weight of insulin (Actrapid; Novo Nordisk). Blood glucose levels were recorded after 15, 30 and 60 min in male H19^∆AT^ (CD, *n* = 5), Control (Control for H19^∆AT^, CD, *n* = 5), H19^∆AT^ (HFD, *n* = 4), Control (Control for H19^∆AT^, HFD, *n* = 5), H19 TG (CD, *n* = 6), Control (Control for H19 TG, CD, *n* = 5), H19 TG (HFD, *n* = 6), and Control (Control for H19 TG, HFD, *n* = 4) mice. At the time of performing glucose tolerance test (GTTs), mice were 13 weeks of age and 10 weeks exposed to CD or HFD feeding. GTT was carried out at 12 am after a 6 h fast starting in the morning. After determining basal blood glucose levels (0 min), animals received an intraperitoneal bolus of 2 g glucose per kilogram of body weight (20% glucose, Delta select). Blood glucose levels were determined 15, 30, 60, and 120 min after injection using an automatic glucose monitor (Contour, Bayer Diabetes Care) in male H19^∆AT^ (CD, *n* = 5), Control (Control for H19^∆AT^, CD, *n* = 5), H19^∆AT^ (HFD, *n* = 4), Control (Control for H19^∆AT^, HFD, *n* = 5), H19 TG (CD, *n* = 6), Control (Control for H19 TG, CD, *n* = 5), H19 TG (HFD, *n* = 5), Control (Control for H19 TG, HFD, *n* = 5) mice. Animals were excluded from analysis that showed no increase/decrease of blood glucose levels after i.p. injection of glucose (GTT) or insulin (ITT), respectively, assuming injection outside of the peritoneal cavity as required for the assay.

### Indirect calorimetry (PhenoMaster)

Upon indirect calorimetry measurements, mice of all genotypes and diets were 16 weeks of age and 13 weeks exposed to CD or HFD. Metabolic measurements were obtained using a PhenoMaster System (TSE Systems). For this, five days before analysis, the mice were placed alone in training cages, identical to the 7.1-l chambers of the PhenoMaster open circuit calorimetry system and continued to receive respective diets (CD, HFD) throughout training and data acquisition. Diets and water were provided ad libitum in the appropriate devices and food intake measured by the built-in automated instruments. Parameters of indirect calorimetry were measured initially for 96 h at 22 °C (warm measurement). Subsequently, temperatures within the PhenoMaster setup were reduced to 4 °C (cold measurement) and data acquired for 96 h. At the end, NE was administered at 4 °C intraperitoneally at a final concentration of 1 mg/kg and measurements continued for another 24 h (NE measurement) to measure maximally activated BAT uncoupling effects. Mean values for each time of day were calculated and plotted for warm, cold and NE measurement in male H19^∆AT^ (CD, *n* = 5), Control (Control for H19^∆AT^, CD, *n* = 5), Male H19^∆AT^ (HFD, *n* = 4), Control (Control for H19^∆AT^, HFD, *n* = 5), H19 TG (CD, *n* = 5), Control (Control for H19 TG, CD, *n* = 4), H19 TG (HFD, *n* = 4), Control (Control for H19 TG, HFD, *n* = 5) mice.

### mRNA isolation and quantitative RT-PCR (qPCR) analysis

Total RNA was isolated from primary adipocytes and tissues using peqGOLD TriFast (PEQLAB Biotechnologie). mRNA was reverse transcribed into complementary DNA using EuroScript reverse transcriptase (Eurogentec). Abundances of mRNAs/lncRNAs were quantified by TaqMan Assay on Demand Kits (Applied Biosystems) according to the manufacturer’s protocol if not indicated otherwise. Abundances of *Adipoq, Afg3l2, CD24, CD29, Cdkn1c, Cebpa, Cebpb, Cox4i1, Cox7a1, Cox8b, Drp1, Elovl3, Fabp4, Fis1, Gata2, H19, hnNctc1, Igf2r, Ly6a, Mff, Mfn1, Mfn2, Mrpl12, Nctc1, Ndufaf2, Ndufaf7, Ndufs2, Ndufv2, Nrf1, Nrf2, Opa1, Peg10, Plagl1, Polg, Ppara, Pparg, Pgc1a, Prdm16, Sdha, Slc22a3, Tgfb2, Ucp1*, and *Uqcr10* were quantified using SYBR methodology using Select Master Mixes (Thermo Fisher). The relative abundance of mRNAs was calculated using comparative methods (2^−δδCT^) according to ABI Relative Quantification Methods. Transcript levels of mRNAs were normalized to hypoxanthine phosphoribosyltransferase 1 (*Hprt1*) expression; *Hprt* abundances were unaffected across all experimental conditions. SYBR primer sequences are provided in Supplementary Data 6.

### Immunohistochemistry

Resected BAT, scWAT, vWAT, and liver specimens were incubated at 4 °C overnight in 4% paraformaldehyde, embedded in paraffin and sliced according to standard protocols. Haematoxylin and eosin (HE) stainings were carried out after deparaffination as described^63^.

### Automated adipocyte quantification

HE stainings were prepared as described above. An automated workflow was devised to segment and measure adipocytes from the tissue images. First, the interactive learning and segmentation toolkit ilastik (www.ilastik.com) was used to train tissue-specific pixel-level classifiers on the RGB photomicrographs. For classification of BAT three classes were defined (membrane, vacuole, nucleus) and a classifier using a set of eleven optimized features selected by the filter method was trained based on a manually labeled training data set. For images from scWAT and vWAT a unified classifier was trained with just two classes (membrane and vacuole) and using all available features. Application of the classifiers to the respective image sets resulted in two or three class probability maps, which were fed in Cellprofiler to segment and individually measure single adipocytes. The vacuole probability map was smoothed and automatically thresholded. The obtained binary vacuole mask was subjected to morphological closing to fill small holes. The result was used to mask the original vacuole probability image, which then was used to perform intensity/probability based cell segmentation by means of the Cellprofiler Identify Primary Objects module. The cell masks were expanded until touching their neighboring cells and cells intersecting with the image border before expansion were excluded. Size and shape parameters of the remaining cells were measured.

### Electron microscopy and mitochondrial morphometry

For the fixation of the tissues the specimen were cut into small (~1 mm^3^) pieces and stored in fresh fixative (10 ml Caco-buffer, 1.6 ml of 25 % Glutardialdehyd, 5 ml of 8% PFA in H2O, filled up to 20 ml with DEPC-treated H2O with pH-value adjusted to 7.3) for 16–24 h at 4 °C. For embedding of the fat tissue samples were washed 4× for 20 min in 0.1 M Caco-buffer (pH = 7.2–7.3) at 4 °C. The samples were stored for 2 h in 2% OsO_4_ in 0.1 M Caco-buffer (pH = 7.2–7.3) at 4 °C in the dark. Afterwards, samples were washed in 0.1 M Caco-buffer (pH = 7.2–7.3) 4× for 20 min at 4 °C. For dehydration samples were stored in ice-cold 50% EtOH for 20 min at 4 °C. Next, samples were kept in ice-cold 70% EtOH at 4 °C overnight. The next day, samples were stored for 20 min in ice-cold 90% EtOH at 4 °C. After that, the samples were stored 3× for 20 min in ice-cold 100% EtOH at 4 °C, 20 min in equal volumes of Propylenoxid:EtOH at 4 °C and 2 × 20 min in pure Propylenoxid at 4 °C. For transfering the samples to epon (20 g Epoxy, 11 g DDSA, 9 g NMA, 0.8 g DMP30) samples were stored for 5 h in equal volumes of Epon:Propylenoxid at 4 °C. Moreover, samples were incubated in 3:1 volumes of Epon:Propylenoxid overnight at 4 °C. Fresh epon was used first to store the samples at 4 °C during the day as well as overnight at 4 °C. On the next day, samples were immersed in fresh epon for 2 h with opened lid. The final embedding was performed in a flat form in fresh epon for 72 h at 62 °C. After embedding the samples, semi-thin sections (500 nm) were cut with a Leica UC6 ultramicrotome to check the quality of the sample. Afterwards, ultrathin sections were produced. The sections were fit to the grid and contrasted. The grid was incubated for 15 min in 1.5% aqueous Uranylacetate, washed 5× in distilled H_2_O, incubated again for 4 min in lead citrate and washed 5× in distilled H_2_O. Finally, samples were checked with electron microscope Jeol 2100 Plus equipped with a Gatan camera.

### Study participants

Paired samples of scWAT and omental vWAT were obtained from 169 individuals (113 women, 56 men). The age ranged from 19 to 88 years and BMIs from 18.9 to 73.1 kg/m². All adipose tissue samples were collected during laparoscopic abdominal surgery^64^. Adipose tissue was immediately frozen in liquid nitrogen and stored at −80 °C. The study was approved by the Ethics Committee of the University of Leipzig (approval no: 159-12-21052012), and performed in accordance to the declaration of Helsinki. All subjects gave written informed consent before taking part in this study. BMI was calculated by weight (kg) divided by square of height (m). All baseline blood samples were collected between 8 and 10 am after an overnight fast. Plasma glucose, HbA1c, HDL-, LDL-cholesterol, free fatty acids, and triglycerides were measured in an automated analyzer (Cobas 8000, Roche Diagnostics, Mannheim, Germany).

### Analysis of H19 expression

RNA from human scWAT and vWAT was extracted by using RNeasy Lipid Tissue Mini Kit (Qiagen, Germany). Quantity and integrity of RNA was monitored via spectrophotometry using NanoVue plus (GE Healthcare, Germany). A volume of 1 µg total RNA from scWAT and vWAT adipose tissue were reverse transcribed with standard reagents (Life technologies, Germany). cDNA was processed for TaqMan probe-based quantitative real-time polymerase chain reaction (qPCR) using the QuantStudio 6 Flex Real-Time PCR System (Life technologies, Germany). Expression of *H19* was calculated by standard curve method and normalized to the expression of hypoxanthine guanine phosphoribosyltransferase 1 (*HPRT1*) as housekeeping gene. TaqMan probes (Life technologies, Germany) for *H19* (Hs00262142_g1) and *HPRT1* (Hs01003267_m1) span exon-exon boundaries to improve the specificity of the qPCR.

### CHART-MS

For ChART-MS, PIBA cells were seeded and either only grown to confluency or induced to differentiate. The cells were washed with 1× PBS and fixed with 1 % Formaldehyde for 10 min and fixation quenched with 0.125 M Glycine. The cells were washed with ice-cold 1× PBS, scraped off and centrifuged for 5 min at 100× *g*. The supernatant was aspirated and cells stored on −80 °C. The cells were lysed in 400 µl lysis buffer, resuspended and stored on ice for 5 min. This step was repeated and 0.1% of NP-40 was added to the lysis buffer. 1 ml of Sucrose buffer was pipetted into a new tube. The lysed cells (max. 400 µl) were carefully pipetted to the center of the sucrose buffer and samples centrifuged for 10 min at 7000× *g* at 4 °C. The lysis step with only 400 µl of lysis buffer and the sucrose step was repeated. After centrifugation, supernatants were discarded and nuclei washed with 1 ml PBS. The tube was centrifuged for 1 min at 7000× *g*, supernatants discarded and 1 ml of PBS added. Suspensions were resuspended and vortexed until achieving a single nucleus suspension. For crosslinking of nuclei 2% formaldehyde was added for 30 min, tubes vortexed and stored for 30 min on a wheel at room temperature. For quenching 0.125 M Glycine was added and samples centrifuged for 1 min at 7000× *g* at 4 °C. Supernatants were discarded and nuclei washed with 1 ml PBS. The samples were centrifuged at 7000× *g* for 1 min. Washing was repeated with 2 min of centrifugation in between. Supernatants were discarded and 300 µl nuclear lysis buffer and RNAse inhibitor added. Samples were vortexed and incubated for 5 min on ice. PBS and protease inhibitor were added in equal volumes to nuclear suspension and the suspensions vortexed. To lyse residual nuclei a tip sonicator was used with the following conditions: 3× cycles 10%, 3 × 10 s, POWER 10%. The suspension was transferred into Diagenode TPX microtubes and tubes sonicated with following conditions: Amplitude: 35%, 15 s ON, 15 s OFF, 36 cycles, time: 9 min for appropriate shearing of mature brown adipocyte chromatin. Afterwards, samples were centrifuged at 17,000× *g* for 5 min and supernatants transferred to new tubes. Gross nucleic acid abundances were quantified using a Nanodrop instrument and 75 µg chromatin mixed with RNAse inhibitor and 650 pmol of oligonucleotides (OGNs) conjugated to Biotin-TEG. (Biotin-TEG increases OGN–biotin distances to ~15 atoms using a triethyleneglycol (TEG) spacer. Biotin-TEG therefore is used to avoid steric hindrances and improve attachment of OGNs to beads.) Samples were vortexed carefully and spun down, stored at 55 °C and shaked on 600 rpm for 10 min. Temperature was decreased to room temperature and samples stored overnight at 55 °C. The beads were mixed with 1 ml of Solution 1 (1 M NaCl and 0.1 % Tween), tubes vortexed, centrifuged shortly and transferred to a magnetic rack. The beads were washed 3× with solution 1. In the meantime, samples were adjusted to 1 M NaCl, vortexed and 50 µl of beads and RNase inhibitor added to the solution. The tubes were stored on a rotator for 30 min at RT, centrifuged, stored in the magnetic rack and supernatants discarded. Beads were washed 2× with 1 ml of Solution 1 and 3× with 1 ml of Solution 2 (1 M NaCl, 0.1% Tween, 0.1 % SDS). For this, solutions were added, tubes vortexed and stored on a shaker 1050 rpm set to 16 °C for 5 min. Afterwards, tubes were stored in the magnetic rack and supernatant discarded. For the final elution, beads were resuspended in 300 µl elution buffer (0.2 M NaCl, 0.1% SDS, 10 mM Tris pH = 8.0) and 1× Proteinase inhibitor. The samples were incubated on a ThermoShaker for 4–5 h and shaken at 1450 rpm at 65 °C. Finally, eluate was concentrated with Amicon Ultra-0,5 Centrifugal filter. To degrade RNA contaminations, samples were treated with RNAse A. Sequences for *H19* sense and antisense oligonucleotides for RNA-immunoprecipitation are provided in Supplementary Data 6.

### Sample preparation for MS

DTT was added at a final concentration of 5 mM and samples were incubated on 55 °C for 30 min. Afterwards, samples were cooled down to room temperature and Cloroacetamid (CAA) added at final concentrations of 40 mM for 30 min in the dark. Protein digestion was performed using the Single-Pot Solid-Phase-enhanced Sample Preparation technology (experimental details available upon request). In brief, 2 µL of a 10 mg/mL mixture of hydrophilic and hydrophobic carboxylate coated paramagnetic beads (SeraMag Speed Beads, Cat No. 44152105050250 and 24152105050250, GE Healthcare) were added to each sample. Acidified acetonitrile was added to achieve a final concentration of 50% organic solvent. Bound proteins were washed with 70 % ethanol and 100 % acetonitrile. Beads were resuspended in 5 µL 50 mM Triethylammoniumbicarbonate buffer containing 0.1 µg Trypsin (Sigma) and 0.1 µg LysC (Wako). Digestion was carried out for 16 h at 37 °C in a PCR cycler to ensure constant temperatures. Recovered peptides were resuspended in 1% formic acid / 5% DMSO and stored at −20 °C prior to MS analysis.

### Mass spectrometry

All samples were analyzed on a Q-Exactive Plus (Thermo Scientific) mass spectrometer that was coupled to an EASY nLC 1000 UPLC (Thermo Scientific). Peptides were loaded with solvent A (0.1% formic acid in water) onto an in-house packed analytical column (50 cm × 75 µm I.D., filled with 2.7 µm Poroshell EC120 C18, Agilent). Peptides were chromatographically separated at a constant flow rate of 250 nL/min using the following gradient: 5–30% solvent B (0. 1% formic acid in 80% acetonitrile) within 119 min, 30–50% solvent B within 19 min, followed by washing and column equilibration. The mass spectrometer was operated in data-dependent acquisition mode. The MS1 survey scan was acquired from 300–1750 m/z at a resolution of 70,000. The top ten most abundant peptides were isolated within a 2 Da window and subjected to HCD fragmentation at a normalized collision energy of 27%. The AGC target was set to 5 × 10E5 charges, allowing a maximum injection time of 55 ms. Product ions were detected in the Orbitrap at a resolution of 17,500. Precursors were dynamically excluded for 20 s.

### Bioinformatic analysis of MS data

All mass spectrometric raw data were processed with Maxquant (version 1.5.3.8) using default parameters. Briefly, MS2 spectra were searched against the Uniprot MOUSE.fasta database, including a list of common contaminants. False discovery rates on protein and PSM level were estimated by the target-decoy approach to 0.01% (Protein FDR) and 0.01% (PSM FDR). Minimal peptide length was set to 7 amino acids and carbamidomethyolation at cysteine residues was considered as a fixed modification. Oxidation (M) and Acetyl (Protein N-term) were included as variable modifications. The match-between runs option was enabled. LFQ quantification was enabled using default settings. The Maxquant output was processed as follows: protein groups flagged as reverse, potential contaminant or only identified by site were removed from the proteinGroups.txt. The remaining table was analyzed with R. Protein groups with at least two valid values out of three replicates (or 2 out of 2) in at least one of the bait subgroups were directed to statistical analysis. The R STATS Package was used to perform Student’s t-tests to obtain significantly changed proteins. Those peptides with *p*-values < 0.05 and log2 fold-changes > 1 were considered as signficantly different. Proteins that provide 2 or 3 valid values in one bait group and zero in the other, were also considered as significantly different.

### Ingenuity pathway analysis and AMIGO2 GO Term Analysis

In total 3590 peptides were detected by MS. All 168 uniquely identified and significantly enriched (see significance criteria above) peptides with fold-changes ≥ 1.5 between *H19* antisense compared to sense oligonucleotide immunoprecipitation in confluent (*n* = 114) or differentiated (*n* = 102) conditions were analyzed using Ingenuity Pathway Analysis. To construct a functionally interconnected *H19* protein interactome, all *n* = 168 gene were loaded into IPA and gene nodes with *n* ≥ 1 edges to other genes retained. For GO Term classification of the remaining *n* = 61 *H19* interaction partners, corresponding Gene Symbols were loaded into AMIGO2 (http://amigo.geneontology.org) and those proteins counted classified by GO Terms RNA binding or Chromatin binding or Chromating modification.

### Analyses of public datasets

PEG and MEG expression in WAT of 24 strains of mice was extracted from publicly available datasets as described by Kraus et al.^65^, Briefly, expression levels of PEGs and MEGs in WAT were obtained from [www.BioGPS.org] - Adipose (MOE430 V2) Obesity-resistant versus obesity-prone is defined based on body composition data from Svenson et al.^66^ and “Naggert1” in the Mouse Phenome Database [www.jax.org/phnome], in which body composition was measured in 43 different mouse stains that were fed a high-fat diet for 8 weeks. Mouse line PL/J is not studied in Naggert1, but its percentage fat weight at 20 months of age is approximately one standard deviation below the average of 32 mouse strains in the Ackert1 data set [www.jax.org/phnome]. PEG and MEG expression was analyzed by GSEA in surgically removed biopsies of WAT of future high and low weight gainers from C57BL6/J mice biopsied prior to treatment with high-fat diet^53^.

### Serum analyses

Serum was obtained by allowing the blood to clot at 4 °C for 4 h. The clotted blood was centrifuged for 10 min at 6200× *g*, the supernatants transferred to a new tube and centrifuged for another 10 min at 17,000× *g*. Serum was collected and stored at −80 °C diluted 1:3 with 0.9% NaCl. For analysis of cholesterole levels the Cholesterin CHOD-PAP (Roche Diagnostics) and to determine trigycerides levels the Triglyceride GPO-PAP of Roche Diagnostics was used. Both analyses were run with the Cobas C 702 (Roche Diagnostics). The analysis was conducted at the Institute for clinical chemistry at the University Clinics in Cologne.

### Pyrosequencing

DNA methylation was analyzed by pyrosequencing. To this end, 1 µg DNA from 1°BAT transfected with siCtrl or siH19 was Bisulfite-converted using the EZ DNA Methylation kit (Zymo), PCR amplified, sequenced on a PyroMark Q96 (Qiagen), and analyzed with the PyroMark CpG SW 1.0 software (Qiagen) as described recently^67^. Primer sequences are provided in Supplementary Data 6.

## Electronic supplementary material

Supplementary Information

Description of Additional Supplementary Files

Supplementary Data 1

Supplementary Data 2

Supplementary Data 3

Supplementary Data 4

Supplementary Data 5

Supplementary Data 6

Supplementary Data 7

## Data Availability

All relevant data are available from the authors. Raw data were deposited within GEO under accession no. GSE116227.
