# Supplementary material for: LincRNA H19 protects from dietary obesity by constraining expression of monoallelic genes in brown fat
Source: Nat Commun. 2018 Sep 6;9:3622. doi: 10.1038/s41467-018-05933-8 (PMC6127097; doi:10.1038/s41467-018-05933-8)
Supplement: Supplementary file 1 — Supplementary Information [file 41467_2018_5933_MOESM1_ESM.pdf]

Supplementary Figure 1

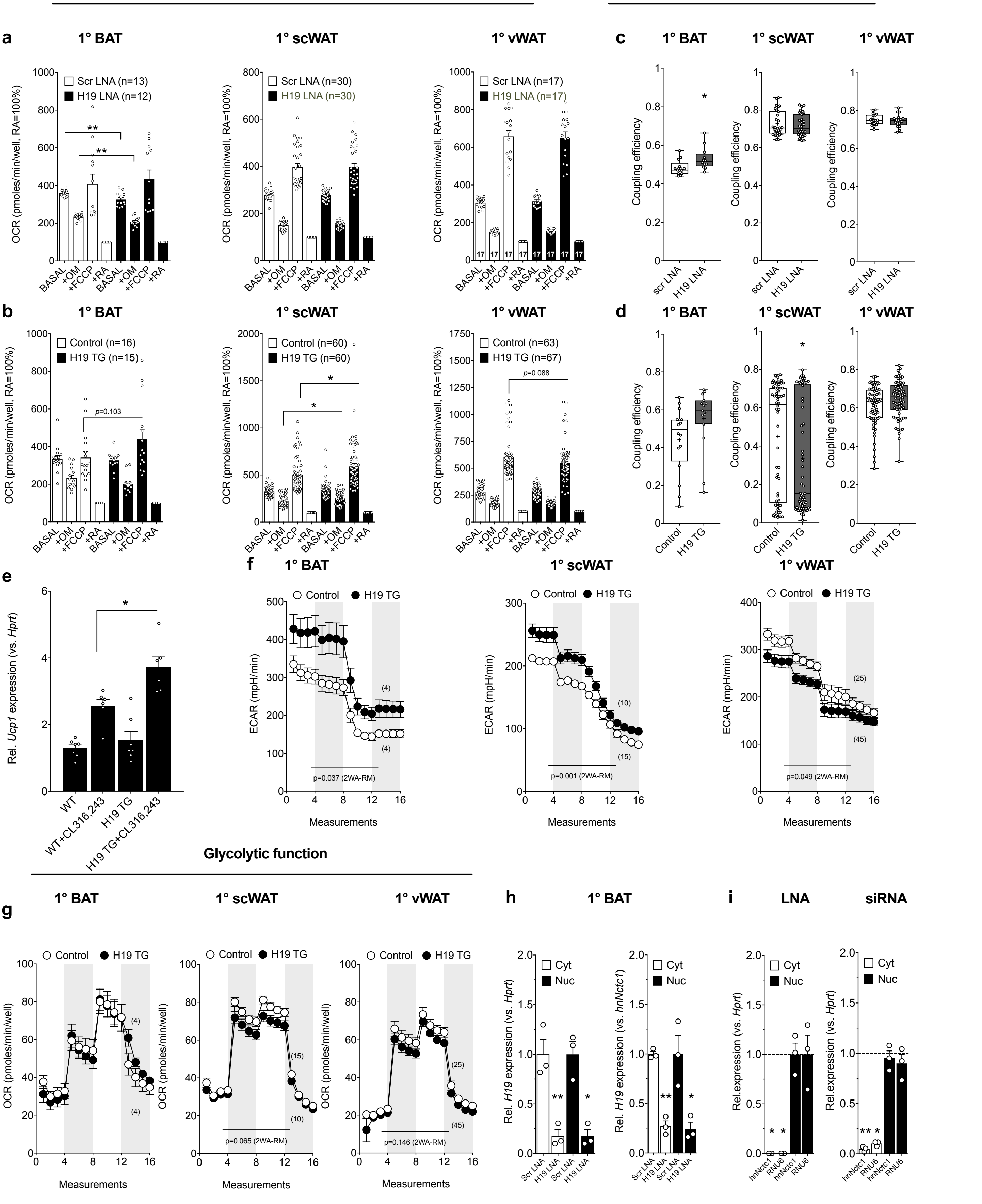

Gene expression analysis, OCR and ECAR quantification in SVF-derived primary (1°) adipocytes from different fat depots after H19 loss- and gain-of-function.
(a) Relative OCRs in BAT *(left)*, scWAT *(middle)* and vWAT *(right)* 1° adipocytes under basal conditions and after addition of oligomycin, FCCP and rotenon and antimycin A transfected with scr or *H19* LNAs. **(b)** Relative OCRs in BAT *(left)*, scWAT *(middle)* and vWAT *(right)* 1° adipocytes isolated from H19 TG or Controls under basal conditions and after addition of oligomycin, FCCP and rotenon and antimycin A. The number of wells measured are indicated in each panel. Bar graphs represent mean ± s.e.m. with all data points plotted and unpaired, two-tailed Student's t-tests were used to assess significance. *p<0.05, **p<0.01, p<0.001. If applicable p-values are indicated within the panel. **(c, d)** Coupling efficiencies calculated from absolute OCRs from Fig.2d,e according to[65]. **(e)** Expression of *Ucp1* in 1°BAT from H19 TG after 6h stimulation with CL316,243. **(f)** ECAR in BAT (*left*), scWAT (*middle*) and vWAT (*right*) 1° adipocytes transfected with scr or *H19* LNA. **(g)** OCRs in BAT (*left*), scWAT (*middle*) and vWAT (*right*) 1° adipocytes transfected with scr or *H19* LNAs. Alternating backgrounds depict injections of medium, glucose, oligomycin and 2-Desoxyglucose. Numbers of measured wells is indicated in brackets for each genotype. A 2WA-RM was applied to assess significance. **(h, i)** *H19* **(h)** and *nuclear transcripts HnNctc1 and RNU6* **(i)** expression in nuclear and cytoplasmic compartments of 1° BAT transfected with *H19* LNA or siH19 inhibitors followed by subcellular fractionation using PARIS kits.

Supplementary Figure 2

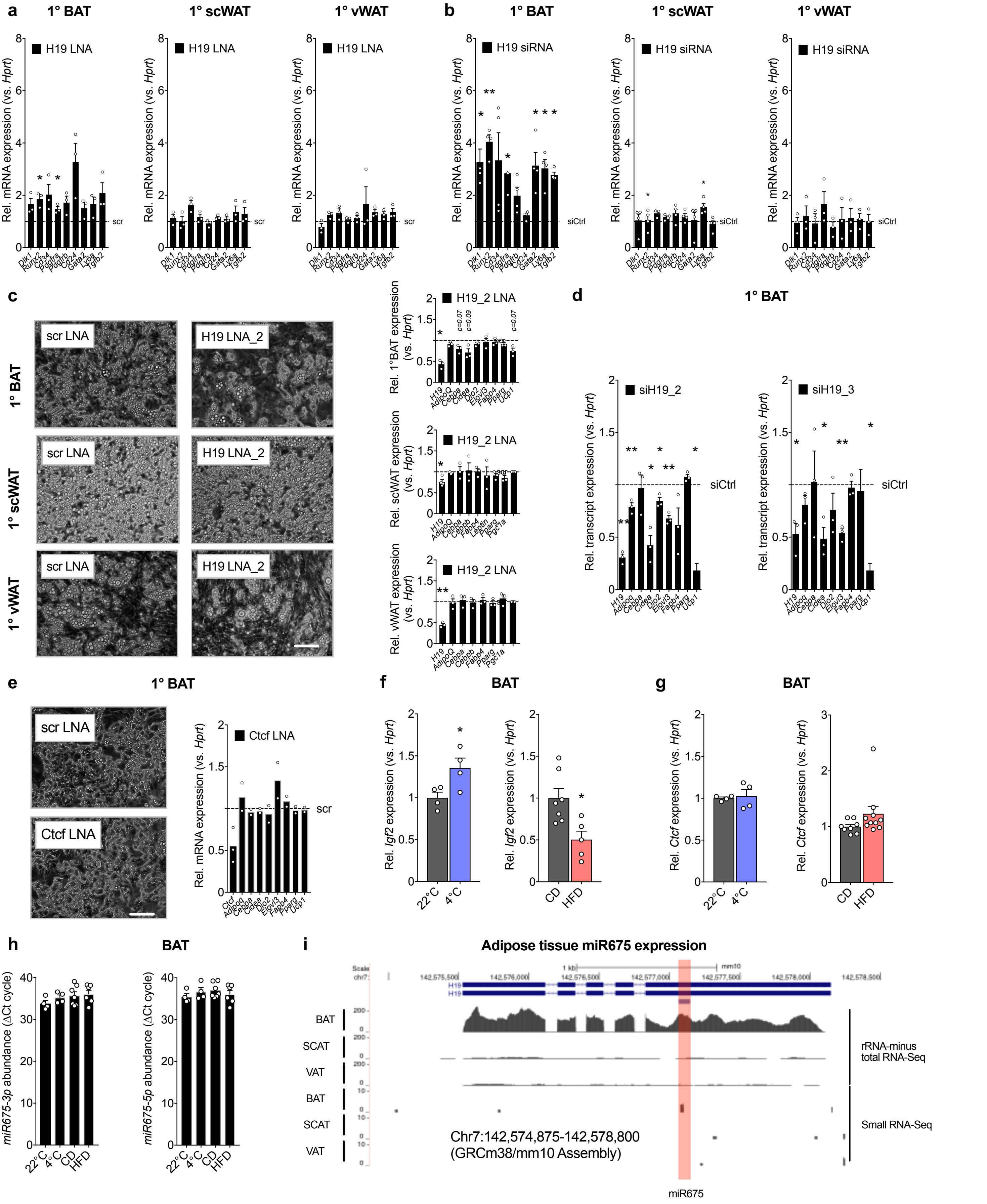

Expression of genes relevant to adipocyte progenitor function, H19-Igf2 imprinting control and H19 function.
(a, b) Expression of indicated mRNAs in BAT, scWAT and vWAT 1° adipocytes transfected with scr or *H19* LNAs. A paired, two-tailed Student's t-test was used to assess significance across n=3 independent experiments, n=3 replicates each. **(c, d)** Photomicrographs **(c)** and gene expression **(c, d)** of indicated mRNAs in BAT, scWAT and vWAT 1° adipocytes transfected with 25nM scr or H19_2 LNAs **(c)** or 100nM siCtrl versus siH19_2 and siH19_3 **(d)**. **(e)** Photomicrograph (*left*) and expression of indicated mRNAs (*right*) in 1° BAT transfected with scr or *Ctcf* LNAs. Pictures are representative of n=2 independent experiments. Gene expression analysis was performed twice, n=3 replicates each. **(c, e)** Scale bar, 100 µm **(f)** *Igf2* expression in lean mice housed at 22° C (n=4) versus 24h of 4° C (n=4, *left*) or exposed to HFD (n=5) versus CD (n=7, *right*) feeding. **(g)** BAT *Ctcf* expression in lean mice housed at 22° C (n=4) versus 24h of 4° C (n=4, *left*) or exposed to HFD (n=10) versus CD (n=8, *right*) feeding. **(h)** *miR675-3p* and *miR675-5p* abundance (ΔCt value) in lean mice housed at 22° C (n=4) versus 24 h of 4° C (n=4) or exposed to HFD (n=6) versus CD (n=7, *right*) feeding. **(i)** Illustration of *H19* (*top*) and *miR675* (*bottom*) abundances in indicated adipose tissues determined by total (*top*) or small RNA-Seq (*bottom*, UCSC Genome Browser). *p<0.05.

Supplementary Figure 3

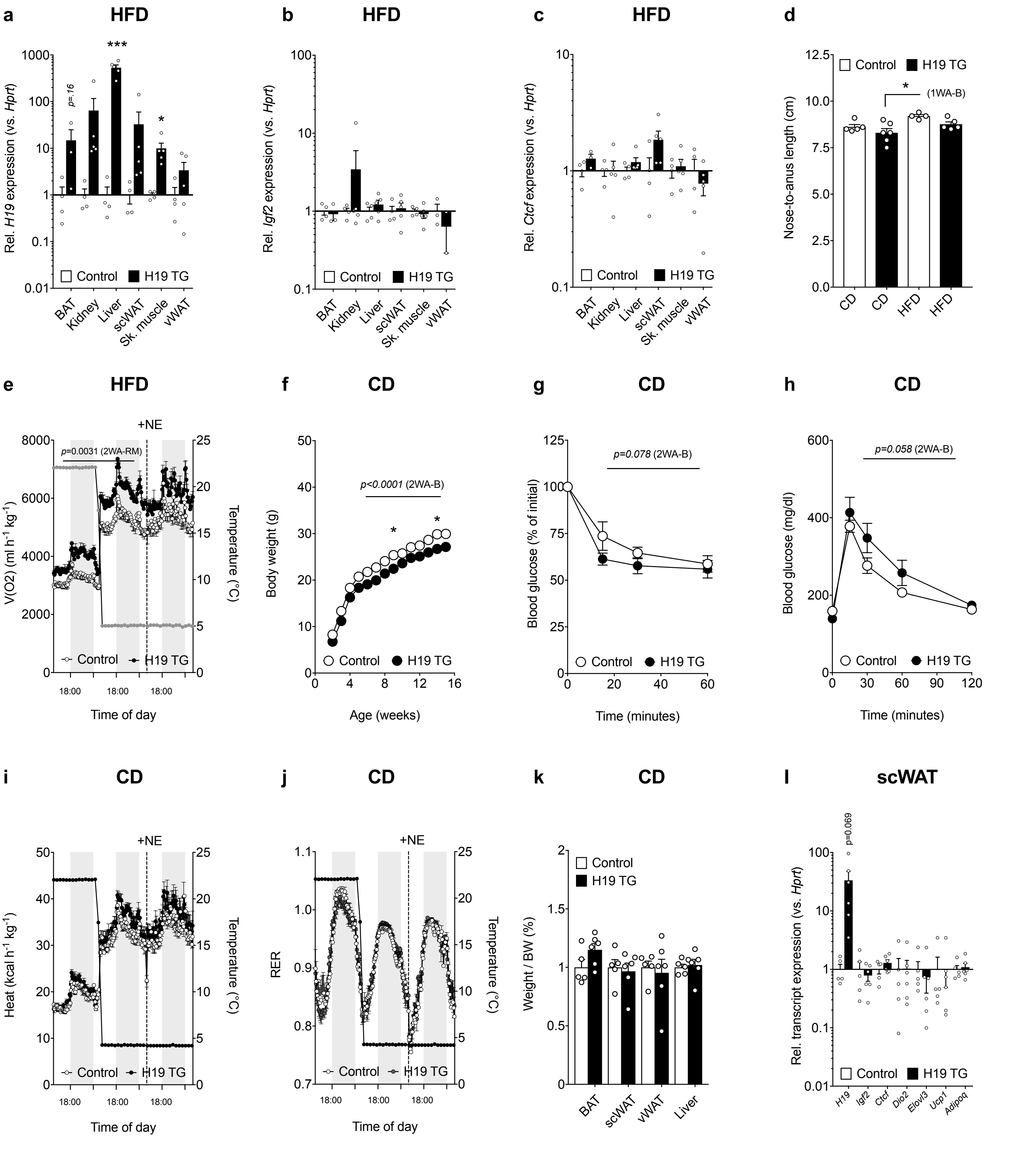

H19 overexpression causes minor increases of energy expenditure in CD-fed H19 TG mice.
(a) *H19* expression in indicated tissues from HFD-fed Control (n=4) versus H19 TG (n=3 for BAT, n=5 for other tissues) mice. **(b)** *Igf2* expression in indicated tissues from HFD-fed Control (n=4) versus H19 TG (n=2 for vWAT, n=3 BAT, n=5 for other tissues) mice. **(c)** *Ctcf* expression in indicated tissues from HFD-fed Control (n=4) versus H19 TG (n=3 BAT, n=5 for other tissues) mice. Bar graphs represent mean ± s.e.m. with all data points plotted and unpaired, two-tailed Student's t-tests were used to assess significance. **(d)** Nose-anus body length in CD-fed Control (n=5) versus H19 TG (n=6) and HFD-fed Control (n=4) versus H19 TG (n=5) mice. Bar graphs represent mean ± s.e.m. with all data points plotted and One-Way ANOVAs with Bonferroni *post* tests were used to assess significance. **(e)** Oxygen consumption in HFD-fed Control (n=4) versus H19 TG (n=5) mice. **(f)** Body weight of CD-fed Control (n=5) versus H19 TG (n=6) mice. **(e, f)** A 2WA-B was applied to assess significance **(g)** Insulin tolerance test of CD-fed Control (n=6) versus H19 TG (n=5) mice. **(h)** Glucose tolerance test of CD-fed Control (n=5) versus H19 TG (n=5) mice. For **(g, h)** a 2WA-B was applied to assess significance. **(i)** Energy expenditure and **(j)** RER in CD-fed Control (n=4) versus H19 TG (n=6) mice. **(k)** Tissue/ body weight ratio in CD-fed Control (n=5) versus H19 TG (n=6) mice. Bar graphs represent mean ± s.e.m. with all data points plotted and unpaired, two-tailed Student's t-tests were used to assess significance. **(l)** scWAT expression of indicated mRNAs from CD-fed Control (n=5) versus H19 TG mice (n=6). An unpaired, two-tailed Student's t-test was applied to assess significance. *p<0.05, ***p<0.001. If applicable p-values are indicated within the panel.

Supplementary Figure 4

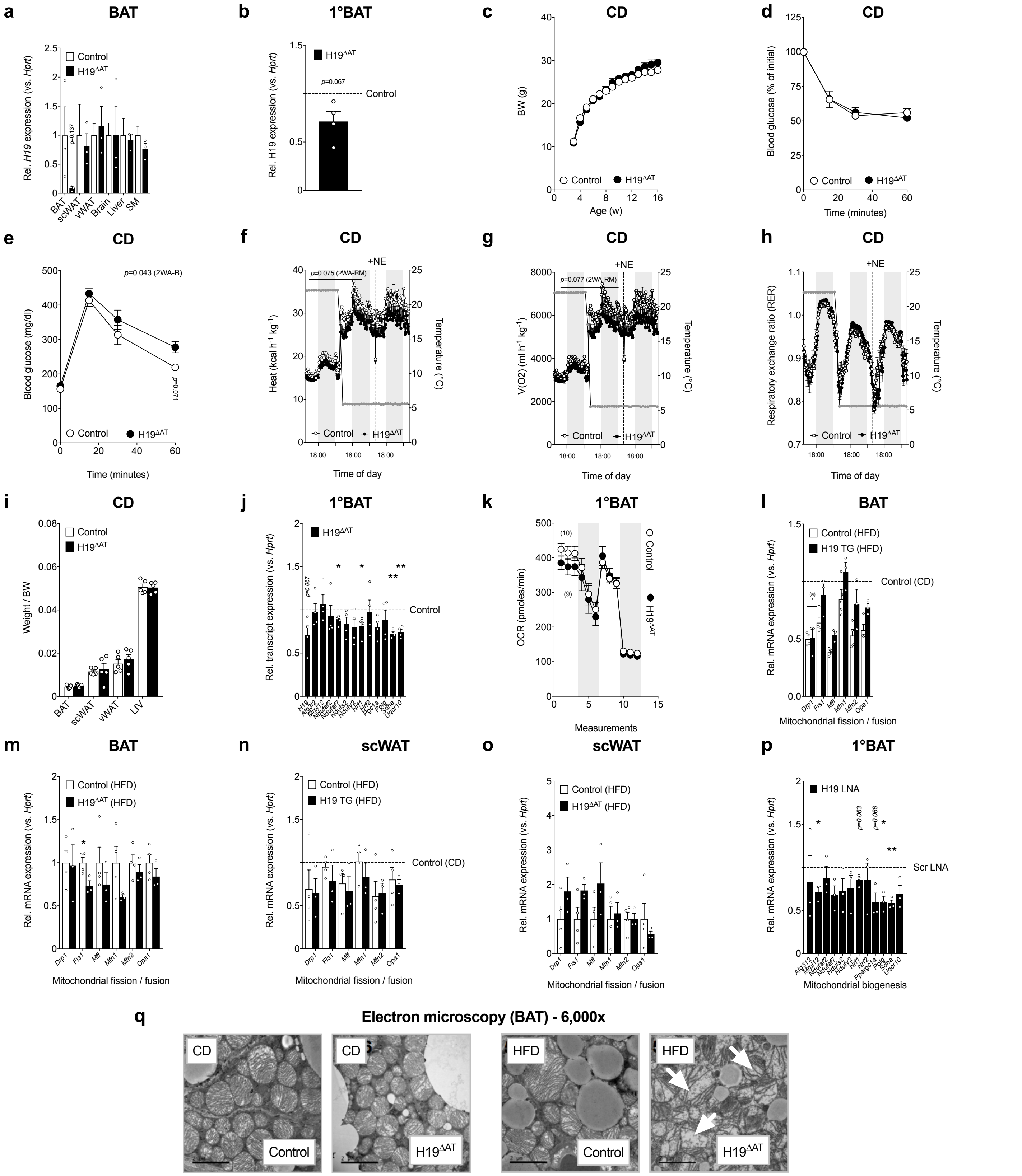

Fat H19 loss causes minor decreases in energy expenditure in lean mice.

(a, b) *H19* expression in indicated tissues from Control (n=3) versus H19$^{\Delta AT}$ (n=3) mice and **(b)** *H19* expression in BAT 1° adipocytes from Control versus H19$^{\Delta AT}$ mice. **(a)** An unpaired, two-tailed Student's t-test was used to assess significant. **(b)** A paired, two-tailed Student's t-tests was used to assess significant across n=4 independent experiments, n=3 replicates each. **(c)** Body weight of CD-fed Control (n=5) versus H19$^{\Delta AT}$ (n=5) mice. **(d)** Insulin tolerance test of CD-fed Control (n=5) versus H19$^{\Delta AT}$ (n=5) mice. **(e)** Glucose tolerance test of CD-fed Control (n=5) versus H19$^{\Delta AT}$ (n=5) mice. Energy expenditure **(f)** oxygen consumption **(g)** and RER **(h)** in CD-fed Control (n=5) versus H19$^{\Delta AT}$ (n=5) mice. **(e-g)** A 2WA-B was applied to assess significance. **(i)** Tissue/body weight ratio in CD-fed Control (n=5) versus H19$^{\Delta AT}$ (n=5) mice. **(j)** Expression of indicated mRNAs in BAT 1° adipocytes from Control or H19$^{\Delta AT}$ mice. A paired, two-tailed Student's t-test was used to assess significance across n=3 independent experiments, n=3 technical each. **(k)** OCRs in 1° BAT from Control or H19$^{\Delta AT}$ mice. Alternating backgrounds depict injections of medium, oligomycin, FCCP and rotenon plus antimycin A. Numbers of measured wells per genotype are indicated. **(l)** BAT expression of indicated mRNAs in HFD-fed H19 TG (n=3), HFD-fed Control (n=4) and CD-fed Control (n=5) male mice. A 1WA-B was applied to assess significance in **(l)**. $^{(a)}$=Significance versus CD-fed Control. **(m)** BAT expression of indicated mRNAs in HFD-fed Control (n=4) versus HFD-fed H19$^{\Delta AT}$ (n=3) mice. **(n)** scWAT expression of indicated mRNAs in HFD-fed H19 TG (n=3), HFD-fed Control (n=4) and CD-fed Control (n=5) mice. **(o)** scWAT expression of indicated mRNAs in HFD-fed Control (n=4) versus HFD-fed H19$^{\Delta AT}$ (n=3) mice. **(p)** Expression of indicated mRNAs in 1° BAT transfected with scr or *H19* LNA. A paired, two-tailed Student's t-test was used to assess significant across n=3 independent experiments performed, n=3 replicates each. *p<0.05, **p<0. 01. If applicable p-values are indicated within the panel. **(q)** Representative electron microscopy images from BAT mitochondria across diets and genotypes with magnifications indicated over the panel. Scale bar, 2 μm.

Supplementary Figure 5

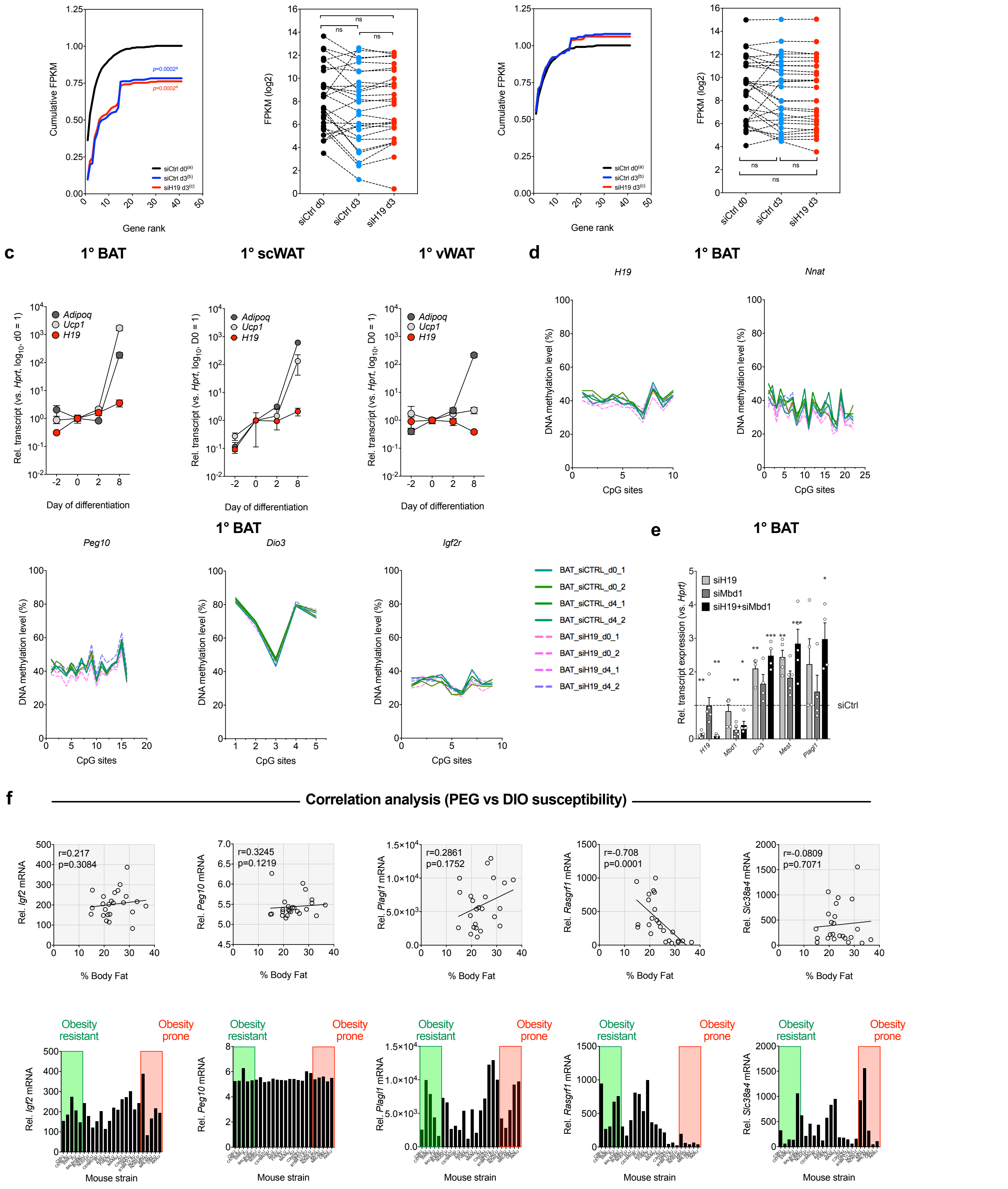

H19 RNAi does not affect MEG expression in BAT and vWAT

(a, b) Cumulative distribution frequency (*left*) and abundances (*right*) of MEGs in **(a)** BAT and **(b)** vWAT 1° adipocytes transfected with siCtrl at d0 (black) versus differentiated siCtrl (blue) and siH19 transfected 1° adipocytes at d3 (red). **(c)** Expression of indicated mRNAs in BAT, scWAT and vWAT 1° adipocytes during the course of differentiation. Data represent 3-4 independent experiments, n=3 replicates each. **(d)** DNA methylation in *H19*, *Nnat* (PEG), *Peg10* (PEG), *Dio3* (PEG) or *Igf2r* (MEG) imprinting control regions of 1°BAT transfected with siH19 or siCtrl. **(e)** Expression of indicated mRNAs in BAT 1° adipocytes transfected with siH19, siMbd1 or combined siH19/Mbd1. An unpaired, two-tailed Student's t-test was used to assess significance. **(f)** Correlation of PEG abundances versus a ranked list of 24 obesity-prone and –resistant non-isogenic mouse strains. PEG abundances were extracted from (MOE430 V2) – Adipose (www.biogps.org[54]) and body composition after HFD feeding defined as 'obesity-resistant' versus 'obesity prone' strains as reported previously[59]. Significance of association between indicated gene expression versus (%) body fat was determined using Spearman's correlation analysis. If applicable additional p-values are indicated within the panel.

Supplementary Figure 6

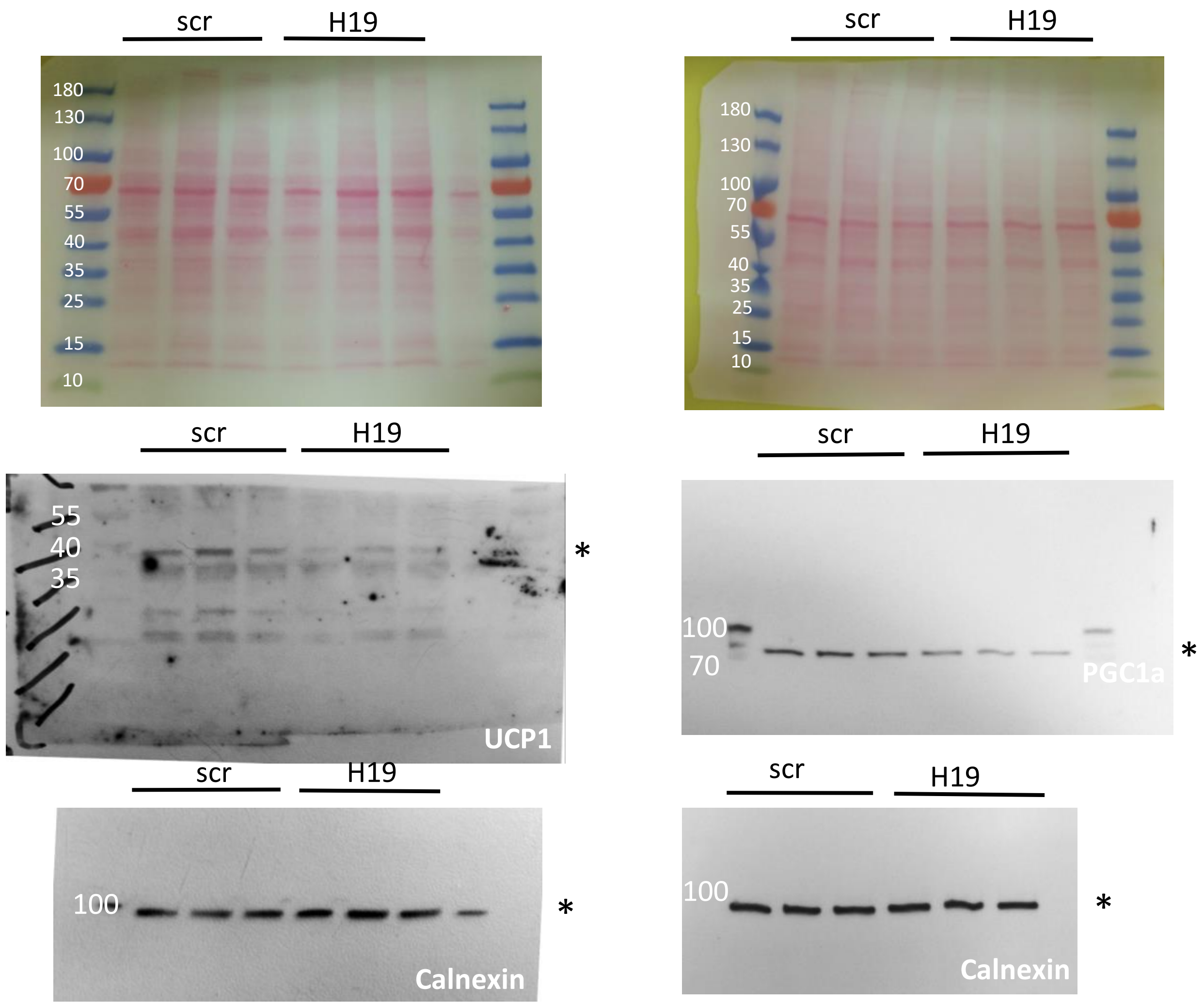

Uncropped images of Immunoblot analyses.
