# Supplementary material for: LincRNA H19 protects from dietary obesity by constraining expression of monoallelic genes in brown fat
Source: Nat Commun. 2018 Sep 6;9:3622. doi: 10.1038/s41467-018-05933-8 (PMC6127097; doi:10.1038/s41467-018-05933-8)
Supplement: Supplementary file 2 — Description of Additional Supplementary Files [file 41467_2018_5933_MOESM2_ESM.docx]

**Description of Additional Supplementary Files**

File Name: Supplementary Data 1

Description:

RNA-Seq of BAT from mice exposed to 4° C for 24 h versus 22° C.

File Name: Supplementary Data 2

Description:

RNA-Seq of BAT from mice subjected to HFD or CD feeding.

File Name: Supplementary Data 3

Description:

RNA-Seq of mature 1°BAT transfected with siH19 or siCtrl siRNAs.

File Name: Supplementary Data 4

Description:

Gene expression in human adipose tissue non-parenchymal cells, mature adipocytes and whole white adipose tissue biopsies.

File Name: Supplementary Data 5

Description:

Correlations of human scWAT and vWAT *hH19* with clinical and gene markers of adipose tissue browning.

File Name: Supplementary Data 6

Description:

Sequence of oligonucleotides used in this study.

File Name: Supplementary Data 7

Description:

Proteins enriched upon *H19* pulldown as revealed by CHART-MS.
